# Analyzing the Effect of Telemedicine on Domains of Quality Through Facilitators and Barriers to Adoption: Systematic Review

**DOI:** 10.2196/43601

**Published:** 2023-01-05

**Authors:** Clemens Scott Kruse, Annamaria Molina-Nava, Yajur Kapoor, Courtney Anerobi, Harshita Maddukuri

**Affiliations:** 1 Texas State University San Marcos, TX United States

**Keywords:** telemedicine, telehealth, mobile health, mHealth, eHealth, quality

## Abstract

**Background:**

Telemedicine has a long history; however, its efficacy has been reported with mixed reviews. Studies have reported a wide range of quality implications when using the telemedicine modality of care.

**Objective:**

This study aimed to analyze the effectiveness of telemedicine through 6 domains of quality through an analysis of randomized controlled trials (RCTs) published in the literature published, to date, in 2022.

**Methods:**

A total of 4 databases were searched using a standard Boolean string. The 882,420 results were reduced to 33 for analysis through filtering and randomization. The systematic literature review was conducted in accordance with the Kruse Protocol and reported in accordance with PRISMA (Preferred Reporting Items for Systematic Reviews and Meta-Analyses; 2020).

**Results:**

The Cohen κ statistic was calculated to show agreement between the reviewers (Cohen κ=0.90, strong). Medical outcomes associated with the telemedicine modality were 100% effective with a weighted average effect size of 0.21 (small effect). Many medical outcomes were positive but not statistically better than treatment as usual. RCTs have reported positive outcomes for physical and mental health, medical engagement, behavior change, increased quality of life, increased self-efficacy, increased social support, and reduced costs. All 6 domains of quality were identified in the RCTs and 4 were identified in 100% of the studies. Telemedicine is highly patient-centered because it meets digital preferences, is convenient, avoids stigma, and enables education at one’s own pace. A few barriers exist to its wide adoption, such as staff training and cost, and it may not be the preferred modality for all.

**Conclusions:**

The effectiveness of telemedicine is equal to or greater than that of traditional care across a wide spectrum of services studied in this systematic literature review. Providers should feel comfortable offering this modality of care as a standard option to patients where it makes sense to do so. Although barriers exist for wide adoption, the facilitators are all patient facing.

**Trial Registration:**

PROSPERO CRD42022343478; https://www.crd.york.ac.uk/prospero/display_record.php?RecordID=343478

## Introduction

### Rationale

The World Health Organization defines telemedicine and telehealth as healing at a distance through the use of information communication technologies to improve health outcomes [1]. The World Health Organization does not distinguish between telemedicine and telehealth; therefore, these terms may be used interchangeably in this study. Mobile health and eHealth enable the practice of medicine and public or population health through mobile devices such as phones, tablets, or patient monitoring devices [2]. Mobile devices have blurred the lines between computers and tablets because the processing power of the 2 have become similar. Many apps work in the same manner on these 2 modalities.

There is no exaggeration to correlate advances in technology with advances in telemedicine. Over the last century, technological advances have connected the world in ways never before thought possible. Once technology enabled communication at a distance, it enabled healing at a distance. The telegraph has even been named the “Victorian Internet” by scholars and was used during the American Civil War to send reports about wounded soldiers to medical teams [3]. Radio and telephone were the next technological advances in communication, and these devices continued the practice of healing at a distance, such as consultations with ships at sea [4]. The modern-day internet and cloud storage have made our world smaller, but the adoption of telemedicine is still not universal.

The COVID-19 pandemic continues to teach the medical community many lessons, but one important lesson is that the modality of telemedicine is possible across a spectrum of services [5] and patients will accept it [6]. For those practices that had not already adopted telemedicine, providers adopted this modality owing to the restriction on face-to-face encounters, and the result was positive; patients were satisfied with the services offered, some providers identified improvements in efficiency, and outcome effectiveness was equally, if not better, than traditional care [6]. However, some providers are still reluctant to adopt telemedicine owing to quality concerns.

Health care quality is a broad but measurable concept. In 1999, the Institute of Medicine defined 6 domains of quality: safe, effective, patient-centered, timely, efficient, and equitable [7]. Safe is avoiding harm. Effective is providing evidence-based care and avoiding the underuse and misuse of medical services. Patient-centered is respecting patient autonomy. Timely is the reduction of wait times. Efficient is the avoidance of waste. Equitable is care that does not vary in the face of personal characteristics [7]. These definitions provide measurable data points.

Telemedicine and its quality have been examined from a specialty point of view, but there has not been a comprehensive look across specialties. Telemedicine has been studied for its quality implications in diabetes [8]; liver disease [9]; pediatrics [10]; gastroenterology [11]; ears, nose, and throat [12]; respiratory care [13]; rheumatoid arthritis [14]; and alcohol use disorder [15]. Each study provides a mix of reviews on quality [16-18].

A systematic review was published in 2020 that examined telemedicine use across multiple service lines in the United States [5]. It analyzed 44 studies over a 5-year period. This review highlighted the agility of the health system of United States in rapidly adopting telemedicine in the face of the pandemic, but it did not report on quality outcomes. It highlighted important concepts for consideration such as risk management, compliance, cost, and patient satisfaction.

A systematic review published in 2022 examined the effect of telemedicine on the quality of care in patients with hypertension and diabetes [19]. This review analyzed 5 studies conducted over 3 years. This review focused on the measurement of effectiveness of medical outcome in the areas of hypertension and diabetes and on patient satisfaction. Telemedicine was found to significantly improve the experience of care and care engagement.

### Objectives

The purpose of this review was to analyze the effectiveness of telemedicine on quality of care across a spectrum of specialties around the world in studies published over the last year, to date, in academic, peer-reviewed journals, using a randomized controlled trial (RCT) or true experiment as the methodology.

## Methods

### Eligibility Criteria

The eligibility criteria for this review were as follows: human participants, published in 2022, published in a peer-reviewed journal, and RCTs, but not reviews. Other systematic reviews were excluded because they had already analyzed studies that could also be included in our review. Their exclusion helped to avoid confounding the results. All reports were in accordance with PRISMA (Preferred Reporting Items for Systematic Reviews and Meta-Analyses) 2020 [20].

### Information Sources

The information sources were PubMed (MEDLINE), CINAHL, Web of Science, and ScienceDirect. The databases were searched on September 19, 2022. These databases were chosen because they were readily available to health care researchers and enabled other researchers to duplicate this study. To eliminate duplicates, MEDLINE was excluded from all databases except PubMed.

### Search Strategy

A Boolean search string was assembled from the keywords provided by the Medical Subject Headings of the United States. Library of Medicine: (tele* OR mhealth) AND (quality OR safe* OR effective* OR timeliness OR “patient centered” OR equitable). The same search string was used for all databases that allowed wildcards. Where wildcards were not allowed, the following search string was used: (telemedicine OR mhealth) AND (quality OR safe OR effective OR timeliness OR “patient centered” OR equitable). Similar filter strategies were used in all databases, because not all databases offered the same filtering tools.

### Selection Process

Following the Kruse protocol, we searched for key terms in all databases, filtered the results, and screened abstracts for applicability [21]. At least two reviewers screened each abstract and analyzed each article. The standard PRISMA diagram was created, as required by the PRISMA standard [20]. Only studies that used the RCT were included in the meta-analysis. Once all filtering and screening were completed, each article was assigned a random number using Microsoft Excel’s random number generator. The first 33 studies were chosen for analysis.

### Data Collection Process

A standardized Excel spreadsheet from the Kruse protocol was used as a data extraction tool to collect additional data at each step of the process [21]. We used a series of 3 consensus meetings to identify articles for full analysis, extract data, and identify themes for analysis.

### Data Items

We collected the following fields of data for each step: Google Scholar search (date of publication, authors, study title, journal, impact factor from Journal Citations Reports, study design, key terms, experimental intervention, results, and comments from each reviewer); filter articles step (the number of results before and after each filter was applied in all 4 databases); abstract screening step (database source, date of publication, authors, study title, journal, screening decision for each reviewer, notes about rejections, consensus meeting one, determination of screening decision, and a set of rejection criteria); analysis step (database source, date of publication, authors, study title, participants, experimental intervention, results compared with a control group, medical outcomes, study design, sample size, bias effect size, country of origin, statistics used, the strength and quality of evidence patient satisfaction, facilitators to adoption, barriers to adoption, and domains of quality). All but the last 4 data items were standard fields on the standardized Microsoft Excel spreadsheet, whereas the last 4 items were specific to the research objective [21].

### Study Risk and Reporting of Bias Assessment

During the data extraction process, reviewers noted individual cases of bias such as sample bias. We combined individual cases of bias with the quality assessment of each study using the Johns Hopkins Nursing Evidence-based Practice (JHNEBP) tool [22]. The strength of evidence was defined by the JHNEBP as level I studies, RCTs or true experiments (with controls and randomization); level II studies, quasi-experimental (control group, but no randomization); level III studies, observational, qualitative, or other nonexperimental methods; and levels IV and V are opinions. Levels IV and V were not considered in this study. We considered instances of bias when interpreting the results because bias can limit external validity [23].

### Effect Measures

Our preferred measure of effect was the Cohen *d*, but other measures were accepted. Measures of effect are summarized in tables for the studies in which they were reported. Measures of effect were reported as Cohen *d*, odds ratios, and β. For studies that reported an effect size, a weighted average effect size was calculated [24]. A Cohen κ statistic was also calculated to measure agreement between reviewers [25,26].

### Synthesis Methods

Reviewers performed a thematic analysis to help make sense of the extracted data [27]. The same or similar observations were consolidated into themes. These themes and individual observations that did not fit into themes were tabulated into affinity matrices for further analysis. The frequency of observations was reported not to imply importance or priority but only to measure the probability of encountering the theme in the group of studies under analysis.

### Additional Analyses and Certainty Assessment

We tabulated the effect sizes during data extraction. Certainty assessments were performed by considering both the narrative analysis and effect size. We calculated the frequency of occurrence of each theme and reported these frequencies in affinity matrices. Frequency reporting provided confidence in the analyzed data.

## Results

### Overview

**Figure 1:**
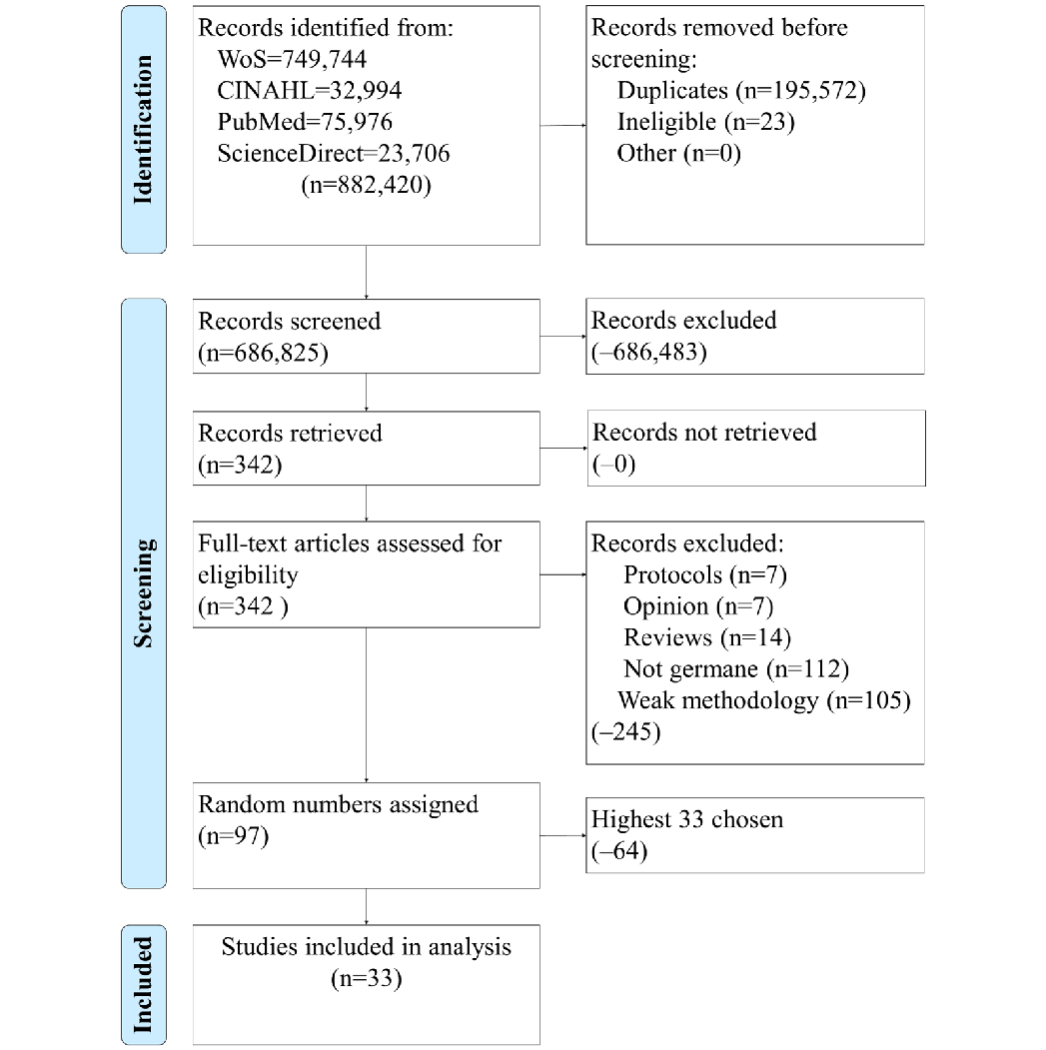
Identification of studies via databases and registries. WoS: Web of Science.

Figure 1 illustrates the study selection process using the PRISMA flow diagram [20]. The query from the 4 databases returned 882,420 results, of which 195,572 were duplicates. The date range and other filters reduced the group to 342 articles for screening. After the screening, 97 studies were included in the analysis. We assigned random numbers to these 97 and chose the highest 33 for data extraction and analysis. Figure 1 also illustrates the articles filtered out for weak methodology if the studies did not use an RCT study design. A ĸ statistic was calculated to reflect the level of agreement between the reviewers (ĸ=0.90, strong agreement) [25,26].

### Study Selection and Characteristics

Following the PRISMA checklist and the Kruse protocol for each study, we extracted the data fields for a Participants, Intervention, Comparison (with the control), Outcome (medical), Study Design table: participants, intervention, comparison (with control or other group), observation, and study design (Table 1). This was performed to summarize the study characteristics in a manner established in the literature. Of the 33 studies analyzed, all were published over a 1-year period [28-60]. Only 6% (2/33) of the studies involved participants younger than 18 years [40,47]. The rest of the studies included participants aged from 18 to 85 years. A total of 5 studies helped participants manage diabetes, 3 helped those living with HIV manage symptoms and relationships, 3 helped participants manage mental health conditions, 2 helped manage hypertension, 2 heart disease, 2 pain management, 2 activity management, and 2 cancer recovery. The rest helped manage tuberculosis, alcohol consumption, smoking cessation, preventive medicine services like vaccinations and tests, stroke recovery, nutrition autism management, Alzheimer disease, and telerehabilitation. More than half (17/33, 52%) of the studies were mobile health, eHealth (10/33, 30%), telephone (3/33, 9%), or general telemedicine (3/33, 9%). All studies demonstrated positive effects, but not all were statistically different from traditional methods of care. The studies originated from 16 countries: China (5/33, 15%), the United States (4/33, 12%), Australia (3/33, 9%), Bangladesh (2/33, 6%), Columbia (2/33, 6%), Germany (2/33, 6%), Japan (2/33, 6%), Spain (2/33, 6%), the United Kingdom (2/33, 6%), Brazil (1/33, 3%), Nepal (1/33, 3%), the Netherlands (1/33, 3%), Norway (1/33, 3%), Sweden (1/33, 3%), Taiwan (1/33, 3%), and Turkey (1/33, 3%).

**Table 1:** PICOS^a^.

Authors	Participants	Experimental intervention (as opposed to traditional care)	Results (compared with control group)	Medical outcomes reported (plainly stated)	Study design
Bao et al [28], 2022	Adults aged 18-60 years treated for TB^b^ in clinic; 57.1% male; 100% Chinese	mHealth^c^ (WeChat) for pulmonary TB self-management	Increase in self-care management behaviors (self-efficacy; *P*<.001), increase in TB knowledge awareness, self-efficacy, social support, and degree of satisfaction with health education (*P*<.001) compared with routine, in-person care in the clinic	Increase in self-efficacy, TB knowledge, social support, and degree of satisfaction with health knowledge	RCT^d^
Bendtsen et al [29], 2022	Adults; average age, 45 years; 58% female	mHealth app for self-reporting of alcohol consumption	Decreased drinking (*P*=.03) more than the control group (traditional counseling); changed behavior	Decreased drinking and changed behavior	RCT
Bhandari et al [30], 2022	Adults with hypertension; average age 50.5 (SD 9.21) years; 44.5% female	mHealth SMS (TEXT4BP) to improve blood pressure	Decreased diastolic BP^e^ (*P*<.001), systolic (*P*<.001) and increase in therapy compliance (*P*<.001), medication adherence (*P*=.02), and knowledge (*P*=.01) over the control (usual treatment)	Decreased BP, increased therapy compliance, increased medication adherence, increase in hypertension knowledge	RCT
Catuara-Solarz et al [31], 2022	Adults with high levels of perceived stress; average age 39.9 (SD 6.11) years; 54% male	mHealth app for mental health	Decrease in anxiety (*P*=.04), resilience (*P*<.001), sleep (*P*=.01), mental well-being (*P*=.02), and stress (*P*=.20) relative to the control group	Decreased anxiety, increased resilience, increased sleep, increased mental well-being, and decreased stress	RCT
Choi et al [32], 2022	Young adult males; average age 21.67 (SD 1.81) years; 74.2% White	Digital HIV intervention (myDex)	Increase in education (*P*=.003), decrease in loneliness (*P*=.004), lower experience of web-based discrimination (*P*=.007), but no difference in behavior at 90-day follow-up; changed dangerous sexual behavior	Increase in education, decrease in loneliness, decrease in web-based discrimination, decreased dangerous sexual behavior but not significantly	RCT
Dalli et al [33], 2022	Adults with acute coronary syndrome; average age 56, (SD 9.4) years; 91.5% male	Cardiac telerehabilitation	Increased mean VO2max^f^ (*P*=.004), decreased apoB/apoA-I^g^ ratio (*P*=.02), increase in physical activity, and return to work was reduced with intervention	Increased VO2max, decrease in apoB/apoA-I, and increase in physical activity	RCT
do Amaral et al [34], 2022	Adults going through smoking cessation; average age 45.7 (SD 12.8) years; 65% female	mHealth SMS for smoking cessation	Costs were lower (*P*<.001) and continuous abstinence reported by both groups	Decreased smoking (continuous abstinence)	RCT
Fernandez et al [35], 2022	Adults calling the 2-1-1 call center for cancer-control and other needs; average age 45.5 (SD 12.4) years; 93.5% female; 43.8% Black	Telephone navigation service	Intervention resulted in greater completion of needed service (*P*=.04), Papanicolaou test (*P*=.02), and smoking cessation (*P*=.04); other areas were improved, but not statistically significant	Greater completion rates, more Papanicolaou tests, greater smoking cessation, completed mammograms, colorectal cancer screening and HPV^h^ vaccinations	RCT
Guillaumier et al [36], 2022	Adult person who has had a stroke; average age 67.5 (SD 12) years; 65% male	eHealth app (Prevent 2nd Stroke, P2S)	QoL^i^ significantly higher for intervention (*P*=.02), reported no problems with personal care (*P*=.04) and usual activities (*P*=.03)	Increased QoL, increased self-efficacy, and increased usual activities	RCT
Gustafson et al [37], 2022	Adults aged >65 years; average age 76.5 (SD 7.4) years; 74% female; 89% White	eHealth app (ElderTree)	Improved depression (OR^j^ –0.20; *P*=.03) and overall mental-health QoL (OR 0.32; *P*=.007) more than the control group	Decreased depression, increased mental health, and increased QoL	RCT
Huggins et al [38], 2022	Older adults recovering from cancer; average age 63.2 (SD 9.9) years; 62% male	Telephone or electronic nutrition counseling	No statistical difference in QALYs^k^ than treatment as usual	Increased QALYs	RCT
Itoh et al [39], 2022	Adults; average age 47.4 (SD 11.3) years; 56.3% male	mHealth app for patient education and strengthening exercise therapy	Intervention group reported less back pain (*P*=.04), higher QoL (*P*=.03), and less fear of movement at week 12 (*P*=.04)	Less back pain, improved QoL, and less fear of movement	RCT
Jamali et al [40], 2022	Children with autism spectrum disorder aged 4-12 years; average age 8.28 (SD 2.57) years; and their parents aged >18 years; average age 37.48 (SD 5.36) years; mostly male	WhatsApp coaching intervention	Intervention group shows greater improvement in occupational performance, specified goals, and behavioral problems	Improved occupational performance, improved specified goals, and improved behavioral problems	RCT
Leong et al [41], 2022	Older adults; average age 58.6 (SD 44.6) years; 68.5% male	Social media–delivered patient education	Change in HbA_1c_^l^ not significant, intervention group showed positive improvements in attitudes (*P*<.001) and self-care activities (*P*=.03); low health literacy contributed to baseline knowledge (*P*=.01)	Improve HbA_1c_, increase in self-efficacy, and increase in attitude	RCT
María Gómez et al [42], 2022	Adults with type 2 diabetes; average age 59.6 (SD 11.7) years; 54.6% male	mHealth app (DM2)	Lower HbA_1c_ levels in intervention group, decreased incidence of hypoglycemia 3.00 mmol/L and severe hypoglycemia	Decreased HbA_1c_, decreased incidence of hypoglycemia and severe hypoglycemia	RCT
Mathiasen et al [43], 2022	Adults; average age 35 (SD 14.1) years; aged 18-71 years; 74% female	Internet-based CBT^m^	Therapy compliance not as statistically high as TAU^n^, decreases in depression not as statistically much as TAU	Maintained therapy compliance and decreased symptoms of depression comparable with treatment as usual	RCT
Molavynejad et al [44], 2022	Adults with type 2 diabetes; average age 47.37 (SD 7.07) years; 50.4% male	Video telecare education	Mean changes of patients’ weight, glycemic parameters, and lipid profiles decreased more in the 2 educational groups than the control group	Lost weight, lower glycemic parameters, and lower lipid profiles	RCT
Morcillo-Muñoz et al [45], 2022	Adults with chronic pain; average age 54.8 (SD 10.7) years; 80% female	Web-based psychosocial chronic pain therapy	Intervention group showed lower catastrophizing (*P*<.001), less helplessness (*P*=.002), improved rumination (*P*<.001), acceptance (*P*=.001), QoL (*P*=.002) over the control; no significant changes reported in magnification and satisfaction with health	Improved catastrophizing, helplessness, rumination, acceptance, and QoL; improvements were also noted in magnification and satisfaction, but these were not statistically significant	RCT
Muschol et al [46], 2022	Adults undergoing follow-up for orthopedic and trauma surgery	Telephone video consultations	The participants from the intervention group reported higher satisfaction, but it was not statistically significant (*P*=.69)	Improved satisfaction	RCT
Nagamitsu et al [47], 2022	Adolescents aged 13-18 years	iCBT^o^	Intervention group reported reduced scores for depressive symptoms and suicide ideation, increase in health promotion, and improved self-monitoring skills to reduce depressive symptoms	Improved depression, less suicide ideation, and more self-efficacy and health promotion	RCT
Ni et al [48], 2022	Adults with coronary heart disease; average age 61 (SD 11) years; 80.1% male	mHealth (WeChat and Message Express) to improve medication adherence	Intervention group showed increase in medication adherence and decrease in systolic BP	Increased medication adherence and decrease in BP	RCT
Pires et al [49], 2022	Adults with type 2 diabetes; average age 43 (SD 8.3) years; 55% female	mHealth app for diabetes management	Intervention group decreased the prevalence of T2DM^p^ and intermediate hyperglycemia	Improved symptoms of T2DM	RCT
Pischke et al [50], 2022	Older adults aged ≥60 and 65-75 years; average age 68.7 years; majority female	eHealth physical activity intervention	Intervention showed increased MVPA^q^	Increased activity	RCT
Roddy et al [51], 2022	Adults with type 2 diabetes; average age 56 (SD 9.5) years; 54% female	mHealth (FAMS^r^) for glycemic control	Family involvement helped decrease HbA_1c_	Decreased HbA_1c_	RCT
Sahin et al [52], 2022	Adults aged ≥60 years who recently underwent knee replacement; average age 66.8 years;	Telerehabilitation for patient with knee replacements	Intervention group demonstrated improvements in movement on the BI^s^ (*P*<.001)	Improved physical function of knee	RCT
Sarker et al [53], 2022	Adults aged >18 years with CKD^t^ ; average age 57.97 (SD 15.03) years; 71% female	mHealth disease education	Intervention group demonstrated lower diastolic BP, lower BMI, and lower salt intake	Improved diet, decreased BMI, reduced BP	RCT
Seib et al [54], 2022	Adults with breast, blood, and gynecologic cancer; average age 52.6 (SD 9.4) years; 100% female; 95% breast cancer	eHealth cancer intervention	Intervention group demonstrated improved general health, bodily pain, vitality, and global physical and mental health scores	Improved physical and mental health, decreased pain, increased vitality	RCT
Skvortsova et al [55], 2022	Adults aged≥18 years; average age 24 (SD 6.79) years	mHealth physical activity intervention	Intervention participants increased daily step count (*P*<.001)	Increased activity	RCT
Stephenson et al [56], 2022	Adult males with HIV; average age 30.4 years; 75% White; 100% male (as assigned at birth)	Telehealth couples counseling and testing	Couples in the intervention group reported safer sexual agreements (*P*=.007), lower odds of discordant relationships (*P*=.048), and lower odds of breaking their sexual agreement (*P*<.001)	Decreased interpersonal problems	RCT
Thesen et al [57], 2022	Adults with noncardiac chest pain; average age 52 years; 54% female	iCBT	Intervention group demonstrated improvements in cardiac anxiety (*P*=.004), and a nonsignificant improvement in fear of bodily sensations (*P*=.07). Improvement in health-related QoL (*P*=.004), increase in physical activity (*P*<.001), improvement in depression (*P*=.03)	Improvement in cardiac anxiety, increased health-related QoL, increased physical activity, improved depression	RCT
Xia et al [58], 2022	Adults with type 2 diabetes; 63.5% male	WeChat+T2DM (TangPlan) to support patients with type 2 diabetes	The intervention group demonstrated improved fasting blood glucose, FBG^u^ (*P*=.048), HbA_1c_ (*P*<.001), body weight (*P*=.006), systolic BP (*P*=.005), diastolic BP (*P*=.03), serum low-density lipoprotein cholesterol (*P*=.006), and cholesterol mean (*P*=.02)	Improved FBG, HbA_1c_, weight, systolic and diastolic BP, serum low-density lipoprotein cholesterol, and cholesterol mean	RCT
Zeng et al [59], 2022	Adults with HIV; 92.3% male (as assigned at birth); 100% Chinese; average age 27.5 years	mHealth WeChat app (Run4Love)	Increased QoL through positive coping (*P*=.006) over control	Increased QoL	RCT
Zhang et al [60], 2022	Adults recovering from cancer; average age 57.6 (SD 12.6) years; 75% male	mHealth questionnaires with follow-up	Intervention group showed fewer irAEs^v^ (*P*=.01), fewer ED^w^ visits (*P*=.01), lower rate of treatment discontinuation (*P*=.02), higher QoL (*P*=.001), and less time implementing follow-up (*P*=.28)	Fewer irAEs, fewer ED visits, better treatment engagement, higher QoL, better follow-up	RCT

^a^PICOS: Participants, Intervention, Comparison (to control), Outcome (medical), Study Design.

^b^TB: tuberculosis.

^c^mHealth: mobile health.

^d^RCT: randomized controlled trial.

^e^BP: blood pressure.

^f^VO2max: maximum oxygen consumption.

^g^apoB/apoA-I: comparison of bad cholesterol with good cholesterol.

^h^HPV: human papillomavirus.

^i^QoL: quality of life.

^j^OR: odds ratio.

^k^QALY: quality-adjusted life year.

^l^HbA_1c_: average blood sugar over last 3 months.

^m^CBT: cognitive behavioral therapy.

^n^TAU: treatment as usual.

^o^iCBT: internet-based, cognitive behavioral therapy.

^p^T2DM: type 2 diabetes mellitus.

^q^MVPA: moderate to vigorous physical activity.

^r^FAMS: family-focused add-on to motivate self-care.

^s^BI: Barthal index.

^t^CKD: chronic kidney disease.

^u^FBG: fasting blood glucose.

^v^irAE: immune-related adverse event.

^w^ED: emergency department.

### Risk of Bias in and Across Studies

The JHNEBP quality assessment tool identified 100% (33/33) of the studies as level I and level A because all but RCTs were screened out. The JHNEBP tool assessed the strength of evidence as levels I to V: I is an RCT or experiment; II is quasi-experimental; III is qualitative or observational; and IV and V are opinion articles. The JHNEBP tool assessed the quality of evidence as A-C: A was defined by consistent results with adequate sample and control sizes (based on a power analysis), definitive conclusions, and consistent recommendations based on extensive literature reviews. Level B was defined by reasonably consistent results, adequate sample and control sizes, definitive conclusions, and recommendations. Level C was defined by little evidence with inconsistent results, insufficient sample sizes, and nondefinitive conclusions.

Reviewers also noted instances of bias, such as sample and selection bias, because these affect external and internal validity, respectively. There were 33 instances of selection bias and 32 of sample bias. Selection bias was identified when samples were taken from one locality, city, or country. Selection bias was identified when the sample comprised a majority of one sex or race.

### Results of Individual Studies

Table 2 summarizes the results of the individual studies through themes. Several themes are repeated in this table because there were multiple observations in the same study that qualified under these themes. For instance, the theme of improved mental health included improvements in anxiety, mental well-being, stress, loneliness, depression, fear, personal satisfaction, helplessness, rumination, acceptance, resilience, and suicidal ideation. Multimedia Appendices 1 and 2 [28-60] provide an observation-to-theme match for all studies. Multimedia Appendix 3 [28-60] provides other data fields collected during the data extraction phase of the systematic literature review.

**Table 2:** Summary of analysis, sorted chronologically.

Authors	Intervention themes	Result theme	Outcome theme	Satisfaction theme	Facilitator theme	Barrier theme	Domain of quality theme
Bao et al [28], 2022	mHealth^a^	Increase in self-efficacy Improved medical engagement Increase in social support	Increase in self-efficacy Improved medical engagement Increase in social support	Satisfied	Patients value technology Convenience Savings in time and mileage Meets a digital preference Education at own pace Effective	Staff training May not be preferred modality	Safe—Avoiding harm Timely—Reduce wait times Effective—Evidence-based Patient-centered—Respect autonomy
Bendtsen et al [29], 2022	mHealth	Changed behavior	Changed behavior	Satisfied	Patients value technology Convenience Savings in time and mileage Meets a digital preference Meets a digital preference Avoids stigma Effective	Cost Staff training May not be preferred modality	Safe—Avoiding harm Timely—Reduce wait times Effective—Evidence-based Efficient—lean Patient-centered—Respect autonomy
Bhandari et al [30], 2022	mHealth	Increase in physical health Increase in physical health Improved medical engagement Improved medical engagement Improved medical engagement Improved medical engagement	Increase in physical health Improved medical engagement Improved medical engagement Improved medical engagement	Satisfied	Patients value technology Convenience Savings in time and mileage Meets a digital preference Education at own pace Effective	Cost Staff training May not be preferred modality	Safe—Avoiding harm Timely—Reduce wait times Effective—Evidence-based Efficient—lean Patient-centered—Respect autonomy
Catuara-Solarz et al [31], 2022	mHealth	Increase in mental health Increase in mental health Increased QoL^b^ Increase in mental health Increase in mental health	Increase in mental health Increase in mental health Increased QoL Increase in mental health Increase in mental health	Satisfied	Effective Patients value technology Savings in time and mileage Meets a digital preference	Cost Staff training	Safe—Avoiding harm Timely—Reduce wait times Effective—Evidence-based Efficient—lean Patient-centered—Respect autonomy
Choi et al [32], 2022	eHealth	Improved medical engagement Increase in mental health Increase in social support Changed behavior	Improved medical engagement Increase in mental health Increase in social support Changed behavior	Satisfied	Effective Convenience Meets a digital preference Avoids stigma	Staff training Low reimbursement Cost May not be preferred modality	Safe—Avoiding harm Timely—Reduce wait times Efficient—lean Effective—Evidence-based Patient-centered—Respect autonomy Equitable—No variance based on personal characteristics
Dalli et al [33], 2022	Telehealth	Increase in physical health Increase in physical health Changed behavior	Increase in physical health Increase in physical health Changed behavior	Satisfied	Effective Patients value technology Convenience Savings in time and mileage Meets a digital preference	Cost Staff training May not be preferred modality	Safe—Avoiding harm Timely—Reduce wait times Effective—Evidence-based Efficient—lean Patient-centered—Respect autonomy
do Amaral et al [34], 2022	mHealth	Reduced costs Changed behavior	Changed behavior	Satisfied	Effective Patients value technology Convenience Savings in time and mileage Meets a digital preference	Cost Staff training May not be preferred modality	Safe—Avoiding harm Timely—Reduce wait times Effective—Evidence-based Efficient—lean Patient-centered—Respect autonomy
Fernandez et al [35], 2022	Telephone	Improved medical engagement Increase in self-efficacy Increase in self-efficacy	Improved medical engagement Increase in self-efficacy Increase in self-efficacy	Satisfied	Effective Patients value personal guidance Convenience	Cost Low reimbursement	Safe—Avoiding harm Timely—Reduce wait times Effective—Evidence-based Efficient—lean Patient-centered—Respect autonomy
Guillaumier et al [36], 2022	eHealth	Increased QoL Increase in self-efficacy Improved medical engagement	Increased QoL Increase in self-efficacy Improved medical engagement	Satisfied	Effective Patients value technology Convenience Savings in time and mileage Meets a digital preference	Cost Staff training May not be preferred modality	Safe—Avoiding harm Timely—Reduce wait times Effective—Evidence-based Efficient—lean Patient-centered—Respect autonomy
Gustafson et al [37], 2022	eHealth	Increase in mental health Increased QoL	Increase in mental health Increased QoL	Not reported	Effective Patients value technology Convenience Savings in time and mileage Meets a digital preference	May not be preferred modality Staff training Low reimbursement Cost	Safe—Avoiding harm Timely—Reduce wait times Effective—Evidence-based Efficient—lean Patient-centered—Respect autonomy
Huggins et al [38], 2022	Telephone	Increased QALYs^c^	Increased QALYs	Not satisfied	Effective Patients value technology Convenience Savings in time and mileage Meets a digital preference Education at own pace	May not be preferred modality Staff training	Safe—Avoiding harm Timely—Reduce wait times Effective—Evidence-based Efficient—lean Equitable—No variance based on personal characteristics Patient-centered—Respect autonomy
Itoh et al [39], 2022	mHealth	Increase in physical health Increased QoL Increase in mental health	Increase in physical health Increased QoL Increase in mental health	Satisfied	Effective Patients value technology Convenience Savings in time and mileage Meets a digital preference Education at own pace	May not be preferred modality Staff training Cost	Safe—Avoiding harm Timely—Reduce wait times Effective—Evidence-based Efficient—lean Patient-centered—Respect autonomy
Jamali et al [40], 2022	mHealth	Increase in physical health Improved medical engagement Increased QoL	Increase in physical health Improved medical engagement Increased QoL	Satisfied	Effective Patients value technology Convenience Savings in time and mileage Meets a digital preference	May not be preferred modality Staff training Cost	Safe—Avoiding harm Timely—Reduce wait times Effective—Evidence-based Efficient—lean Patient-centered—Respect autonomy
Leong et al [41], 2022	mHealth	Increase in physical health Increase in self-efficacy Increased QoL	Increase in physical health Increase in self-efficacy Increased QoL	Not reported	Effective Patients value technology Convenience Savings in time and mileage Meets a digital preference	May not be preferred modality Staff training Cost	Safe—Avoiding harm Timely—Reduce wait times Effective—Evidence-based Efficient—lean Patient-centered—Respect autonomy
María Gómez et al [42], 2022	mHealth	Increase in physical health Increase in physical health Increase in physical health	Increase in physical health Increase in physical health Increase in physical health	Satisfied	Effective Patients value technology Convenience Savings in time and mileage Meets a digital preference	May not be preferred modality Staff training Cost	Safe—Avoiding harm Timely—Reduce wait times Effective—Evidence-based Efficient—lean Patient-centered—Respect autonomy
Mathiasen et al [43], 2022	eHealth	Improved medical engagement Increase in mental health	Improved medical engagement Increase in mental health	Satisfied	Effective Patients value technology Convenience Savings in time and mileage Meets a digital preference	May not be preferred modality Staff training	Safe—Avoiding harm Timely—Reduce wait times Effective—Evidence-based Efficient—lean Patient-centered—Respect autonomy
Molavynejad et al [44], 2022	eHealth	Increase in physical health	Increase in physical health	Satisfied	Effective Patients value technology Convenience Savings in time and mileage Meets a digital preference Education at own pace	May not be preferred modality Staff training	Safe—Avoiding harm Timely—Reduce wait times Effective—Evidence-based Efficient—lean Patient-centered—Respect autonomy
Morcillo-Muñoz et al [45], 2022	eHealth	Increase in mental health Increased QoL	Increase in mental health Increased QoL	Not satisfied	Effective Patients value technology Convenience Savings in time and mileage Meets a digital preference	Cost Staff training May not be preferred modality	Safe—Avoiding harm Timely—Reduce wait times Effective—Evidence-based Efficient—lean Patient-centered—Respect autonomy
Muschol et al [46], 2022	Telephone	Improved medical engagement	Improved medical engagement	Satisfied	Effective Patients value technology Convenience Savings in time and mileage Meets a digital preference	Cost Staff training May not be preferred modality	Safe—Avoiding harm Timely—Reduce wait times Effective—Evidence-based Efficient—lean Patient-centered—Respect autonomy
Nagamitsu et al [47], 2022	eHealth	Increase in mental health Increase in physical health Increase in self-efficacy	Increase in mental health Increase in physical health Increase in self-efficacy	Satisfied	Effective Patients value technology Convenience Savings in time and mileage Meets a digital preference	Cost Staff training May not be preferred modality	Safe—Avoiding harm Timely—Reduce wait times Effective—Evidence-based Efficient—lean Patient-centered—Respect autonomy
Ni et al [48], 2022	mHealth	Improved medical engagement Increase in physical health	Improved medical engagement Increase in physical health	Satisfied	Effective Patients value technology Convenience Savings in time and mileage Meets a digital preference	Cost Staff training May not be preferred modality	Safe—Avoiding harm Timely—Reduce wait times Effective—Evidence-based Efficient—lean Patient-centered—Respect autonomy
Pires et al [49], 2022	mHealth	Increase in physical health	Increase in physical health	Not reported	Effective Patients value technology Convenience Savings in time and mileage Meets a digital preference	Cost Staff training May not be preferred modality	Safe—Avoiding harm Timely—Reduce wait times Effective—Evidence-based Efficient—lean Patient-centered—Respect autonomy
Pischke et al [50], 2022	eHealth	Changed behavior	Changed behavior	Satisfied	Effective Patients value technology Convenience Savings in time and mileage Meets a digital preference	Cost Staff training May not be preferred modality	Safe—Avoiding harm Timely—Reduce wait times Effective—Evidence-based Efficient—lean Patient-centered—Respect autonomy
Roddy et al [51], 2022	mHealth	Increase in physical health	Increase in physical health	Not reported	Effective Patients value technology Convenience Savings in time and mileage Meets a digital preference	Cost Staff training May not be preferred modality	Safe—Avoiding harm Timely—Reduce wait times Effective—Evidence-based Efficient—lean Patient-centered—Respect autonomy
Sahin et al [52], 2022	Telehealth	Increase in physical health	Increase in physical health	Not reported	Effective Patients value technology Convenience Savings in time and mileage Meets a digital preference Education at own pace	Cost Staff training May not be preferred modality	Safe—Avoiding harm Timely—Reduce wait times Effective—Evidence-based Efficient—lean Patient-centered—Respect autonomy
Sarker et al [53], 2022	mHealth	Increase in physical health Changed behavior	Changed behavior Increase in physical health	Not reported	Effective Patients value technology Convenience Savings in time and mileage Meets a digital preference Education at own pace	Cost Staff training May not be preferred modality	Safe—Avoiding harm Timely—Reduce wait times Effective—Evidence-based Efficient—lean Patient-centered—Respect autonomy
Seib et al [54], 2022	eHealth	Increase in physical health Increase in physical health Increased QoL Increase in mental health	Increase in physical health Increase in physical health Increased QoL Increase in mental health	Not reported	Effective Patients value technology Convenience Savings in time and mileage Meets a digital preference	Cost Staff training May not be preferred modality	Safe—Avoiding harm Timely—Reduce wait times Effective—Evidence-based Efficient—lean Patient-centered—Respect autonomy
Skvortsova et al [55], 2022	mHealth	Increase in physical health Changed behavior	Increase in physical health Changed behavior	Not reported	Effective Patients value technology Convenience Savings in time and mileage Meets a digital preference	Cost Staff training May not be preferred modality	Safe—Avoiding harm Timely—Reduce wait times Effective—Evidence-based Efficient—lean Patient-centered—Respect autonomy
Stephenson et al [56], 2022	Telehealth	Changed behavior Increased QoL	Increased QoL Changed behavior	Satisfied	Effective Patients value technology Convenience Savings in time and mileage Meets a digital preference	Cost Staff training May not be preferred modality	Safe—Avoiding harm Timely—Reduce wait times Effective—Evidence-based Efficient—lean Patient-centered—Respect autonomy
Thesen et al [57], 2022	eHealth	Increase in physical health Increase in mental health Increased QoL	Increase in physical health Increase in mental health Increased QoL	Satisfied	Effective Patients value technology Convenience Savings in time and mileage Meets a digital preference	Cost Staff training May not be preferred modality	Safe—Avoiding harm Timely—Reduce wait times Effective—Evidence-based Efficient—lean Patient-centered—Respect autonomy
Xia et al [58], 2022	mHealth	Increase in physical health Changed behavior	Increase in physical health Changed behavior	Satisfied	Effective Patients value technology Convenience Savings in time and mileage Meets a digital preference	Cost Staff training May not be preferred modality	Safe—Avoiding harm Timely—Reduce wait times Effective—Evidence-based Efficient—lean Patient-centered—Respect autonomy
Zeng et al [59], 2022	mHealth	Increased QoL Changed behavior	Increased QoL Changed behavior	Satisfied	Effective Patients value technology Convenience Savings in time and mileage Meets a digital preference	Cost Staff training May not be preferred modality	Safe—Avoiding harm Timely—Reduce wait times Effective—Evidence-based Efficient—lean Patient-centered—Respect autonomy
Zhang et al [60], 2022	mHealth	Fewer irAEs^d^ Changed behavior Improved medical engagement Increased QoL Improved medical engagement	Fewer irAEs Changed behavior Improved medical engagement Increased QoL Improved medical engagement	Not reported	Effective Patients value technology Convenience Savings in time and mileage Meets a digital preference	Cost Staff training May not be preferred modality	Safe—Avoiding harm Timely—Reduce wait times Effective—Evidence-based Efficient—lean Patient-centered—Respect autonomy

^a^mHealth: mobile health.

^b^QoL: quality of health.

^c^QALY: quality-adjusted life year.

^d^irAE: immune-related adverse event.

### Results of Syntheses, Additional Analysis, and Certainty of Evidence

#### Overview

Thematic analysis was performed to help make sense of the data collected. Themes and individual observations that did not fit the themes were tabulated. The mean sample size was 351.7 (SD 501.1). A total of 11 studies reported the effect sizes [29,31,35-37,41,51,54-57]. Odds ratios were converted to Cohen *d* and a weighted average effect size was calculated (0.21, small effect) [61,62].

#### Results of Telemedicine and Quality

Table 3 summarizes the results. The reviewers identified 7 themes and 3 individual observations for 92 occurrences in the literature. As these were the result of a thematic analysis, there are cases of multiple observations of the same theme in the same article; therefore, the frequency may not have aligned with the number of references. Of the 92, there were 31 (34%) instances of an increase in physical health [30,33,39-42, 44,47,49,51-55,57,58]. This theme included maximum oxygen consumption, comparison of bad cholesterol with good cholesterol, pain, diastolic blood pressure, systolic blood pressure, hypoglycemia, lipids, overall blood pressure, average blood sugar over last 3 months, physical function, fasting blood glucose, cholesterol, and BMI. There were 17% (16/92) of instances of increased mental health [31,32,37,39,43,45,47, 54,57]. This theme encompassed anxiety, well-being, stress, loneliness, depression, fear, personal satisfaction, helplessness, rumination, acceptance, suicidal ideation, and resilience. There were 13% (12/92) of instances of improved medical engagement [28,30,32,35,36,40,43,46,48,60]. This theme included medication compliance, program or treatment adherence, follow-up visits, medical knowledge, and decrease in emergency department visits. There were 12% (11/92) of instances of 2 themes: changed behavior, which included sexual behavior, self-care, drinking, smoking, physical activity, weight loss, and salt intake [29,32,34,50,53,55,56,58-60] and increased quality of life (QoL), which included sleep, vitality, interpersonal problems, attitude, or as measured by the EuroQoL visual analog scale [31,36,37,39-41,45,54,56,57,59,60]. There were 7% (6/92) of instances of increased self-efficacy [28,35,36,41,47]. This theme included an increase in self-care, vaccinations, and preventive measures, such as Papanicolaou smears, colorectal exams, and mammograms. There were 2 instances of increased social support, which included a reduction in web-based discrimination [28,32]. A total of 3 observations did not fit any themes and each occurred only once in the literature: fewer immune-related adverse events (for cancer patients), increased quality-adjusted life years, and reduced costs [34,38,60].

**Table 3:** Results of telemedicine and quality.

Results themes	Frequency (n=92)
Increase in physical health^a^ [30,33,39-42,44,47,49,51-55,57,58]	31
Increase in mental health^a^ [31,32,37,39,43,45,47,54,57]	16
Improved medical engagement^a^ [28,30,32,35,36,40,43,46,48,60]	12
Changed behavior^a^ [29,32,34,50,53,55,56,58-60]	11
Increased QoL^b^ [31,36,37,39-41,45,54,56,57,59,60]	11
Increase in self-efficacy [28,35,36,41,47]	6
Increase in social support [28,32]	2
Fewer irAEs^c^ [60]	1
Increased QALYs^d^ [38]	1
Reduced costs [34]	1

^a^Multiple occurrences were observed in one study.

^b^QoL: quality of life.

^c^irAE: immune-related adverse event.

^d^QALY: quality-adjusted life year.

#### Medical Outcomes of Telemedicine and Quality

Table 4 summarizes the observed medical outcomes. The reviewers identified 7 themes and 2 individual observations for 86 occurrences in the literature. The results compared with the control group and the medical outcomes were highly similar, but they focused on themes and observations relevant to the provider. Some results did not directly correlate with medical outcomes; therefore, the numbers were not exactly the same.

**Table 4:** Medical outcomes of telemedicine and quality.

Outcome themes	Frequency (n=86)
Increase in physical health^a^ [30,33,39-42,44,47,49,51-55,57,58]	29
Increase in mental health^a^ [31,32,37,39,43,45,47,54,57]	16
Improved medical engagement^a^ [28,30,32,35,36,40,43,46,48,60]	11
Increased QoL^a,b^ [31,36,37,39-41,45,54,56,57,59,60]	11
Changed behavior [29,32-34,50,53,55,56,58-60]	10
Increase in self-efficacy [28,35,36,41,47]	6
Increase in social support [28,32]	2
Fewer irAEs^c^ [60]	1
Increased QALYs^d^ [38]	1

^a^Multiple occurrences were observed in one study.

^b^QoL: quality of life.

^c^irAE: immune-related adverse event.

^d^QALY: quality-adjusted life year.

#### Satisfaction Associated With the Adoption of Telemedicine

A total of 24 studies reported on satisfaction. Of the 33 studies, 22 (67%) reported satisfaction or high satisfaction [28-36,39,40,42-44,46-48,50,56-59], 2 (6%) reported dissatisfaction [38,45], and 9 (27%) did not report satisfaction or dissatisfaction [37,41,49,51-55,60].

#### Facilitators to the Adoption of Telemedicine and Quality Implications

Table 5 summarizes the observed facilitators. The reviewers identified 7 themes and 1 individual observation for 166 occurrences in the literature. All 33 (100%) studies reported that the intervention was an effective as modality of treatment [28-36,38-55,57-60]. A digital preference was observed in 97% (32/33) of studies [28-34,36-60]. Convenience was observed in 94% (31/33) of studies [28-30,32-60]. The authors noted the convenience of telemedicine and its ability to be available anytime, anywhere. Telemedicine patients did not need to take off work, drive to a clinic, find a place to park, wait for appointments, and personally expose themselves to the germs in the medical environment [28-31,33,34,36-60]. These savings in time and mileage were observed in 91% (30/33) of studies. In addition, some patients preferred a technologically oriented mode of care or delivery [28-31,33,34,36-60]. Patients valued technology and saved time and mileage in 91% (30/33) of studies. Many studies involved an educational dimension to the intervention. Patients appreciated the telemedicine modality for medical education because it allowed them to absorb or learn at their own pace. This was observed in 21% (7/33) of studies [28,30,38,39,44,52,53]. In 6% (2/33) of studies, one on alcohol consumption and one on HIV management, avoidance of stigma was mentioned [29,32]. Finally, patients valued the personal guidance of a telephone navigator. This was observed in 3% (1/33) of studies [35].

**Table 5:** Facilitators to the adoption of telemedicine and quality implications.

Facilitator themes	Frequency (n=166)
Effective [28-36,38-55,57-60]	33
Meets a digital preference [28-34,36-60]	32
Convenience [28-30,32-60]	31
Patients value technology [28-31,33,34,36-60]	30
Savings in time and mileage [28-31,33,34,36-60]	30
Education at own pace [28,30,38,39,44,52,53]	7
Avoids stigma [29,32]	2
Patients value personal guidance [35]	1

#### Barriers to the Adoption of Telemedicine and Quality Implications

Table 6 summarizes the observed barriers. The reviewers identified 4 themes for 93 occurrences in the literature. Of the 33 studies, the need for staff training appeared in 94% (31/33) of the studies [28-34,36-60]. Thus, telemedicine may not be the preferred modality of care. This was observed in 91% (30/33) of the studies [28-30,32-34,36-60]. The cost of acquiring the servers to manage telemedicine, apps on mobile and computer platforms, and phones themselves were significant barriers. This was observed in 88% (29/30) of the studies [29-37,39-42,45-60]. Finally, in countries where reimbursement was an issue, the rate was lower for telemedicine than for traditional modalities of care. This was observed in 9% (3/33) of the studies [32,35,37].

**Table 6:** Facilitators to the adoption of telemedicine and quality implications.

Barrier themes	Frequency (n=93)
Staff training [28-34,36-60]	31
May not be preferred modality [28-30,32-34,36-60]	30
Cost [29-37,39-42,45-60]	29
Low reimbursement [32,35,37]	3

#### Domains of Quality Incident to the Adoption of Telemedicine

Table 7 summarizes the domains of quality observed in the adoption of telemedicine. Of the 6 domains of quality, 4 observed in all (33/33, 100%) the studies: safe, effective, patient-centered, and timely [28-60]. Efficient was identified in 97% (32/33) of the studies [29-60]. Equitable was only identified in 6% (2/33) of the studies because of the digital divide that often falls on socioeconomic lines [32,38]. The results of these 2 studies were collected and reported on racial and socioeconomic lines.

**Table 7:** Domains of quality incident to the adoption of telemedicine.

Quality themes	Frequency (n=166)
Safe—Avoiding harm [28-60]	33
Effective—Evidence-based [28-60]	33
Patient-centered—Respect autonomy [28-60]	33
Timely—Reduced wait times [28-60]	33
Efficient—lean [29-60]	32
Equitable—No variance based on personal characteristics [32,38]	2

## Discussion

### Summary of Evidence

Commensurate with the objective statement, this systematic literature review analyzed 33 RCT studies from 16 countries published in 2022, to date, to analyze the effectiveness (weighted average effect size 0.21, small) of telemedicine through the lens of 6 domains of quality. All these 33 studies reported the positive effectiveness of telemedicine as a modality across all 6 domains of quality. These studies showed positive outcomes in physical [30,33,39-42,44,47,49,51-55,57,58] and mental health [31,32,37,39,43,45,47,54,57], medical engagement [28,30,32,35,36,40,43,46,48,60], changed behavior [29,32,34, 50,53,55,56,58-60], increased QoL [31,36,37,39-41,45,54, 56,57,59,60], increased self-efficacy [28,35,36,41,47], increased social support [28,32], and reduced costs [34].

Patient engagement is important because it plays a central role in patient safety, chronic disease self-management, adverse event reporting, and medical record accuracy [63]. It also affects health literacy and shared decision-making [64]. Changing patients’ behavior is difficult, and advances in this area often require motivational interviewing [65]. Leveraging telemedicine to increase shared decision-making contributed to behavioral changes in about a third of the studies analyzed. An increase in health-related QoL was also an important conclusion. This facet of health care has become especially important during the COVID-19 pandemic [66]. Finally, leveraging telemedicine to reduce the cost burden is commensurate with other literature [67]. Telemedicine reduces miles driven, time taken off work, and childcare expenses, while maintaining high-quality outcomes [67].

Telemedicine was effective for patients. Studies reviewed in this study mentioned that it is effective [28-36,38-55,57-60], and it meets the digital preference of patients [28-34,36-60] because many patients value technology [28-31,33,34,36-60]. The pandemic has taught health care that telemedicine increases patients’ perception of the availability of care and most patients prefer this modality [68]. It is convenient, saves time and mileage [28-31,33,34,36-60], enables education at one’s own pace [28,30,38,39,44,52,53], avoids stigma [29,32], and provides personal navigation through the health care system. These results serve as strong facilitators for the adoption of telemedicine because they show strong quality results in favor of patient commensurate with other published literature [6].

There are several barriers to telemedicine adoption. Staff must be trained in delivering care through telemedicine to ensure that quality does not decline [28-34,36-60]. Patients must be asked if telemedicine is acceptable because it may not be their preferred modality of care [28-30,32-34,36-60]. Hardware and software costs are associated with enabling this modality care [29-37,39-42,45-60]. The cost of telemedicine infrastructure is consistent with published literature [6]. Finally, in countries where reimbursement remains a consideration, there are low rates of reimbursement for this modality of care [32,35,37]. These results serve as barriers to the adoption of telemedicine, which can be addressed through policies and incentives.

Of the 6 domains of quality, 4 (67%) were identified in all of the analyzed studies: safe, effective, patient-centered, and timely. Efficiency was only mentioned in 97% (32/33) of studies and equitable in only 6% (2/33) of studies. This is largely owing to the technology gap that occurs along socioeconomic lines. This disparity has been identified in other literature [69]. Identifying all 6 domains of quality in the literature also serves as a strong indicator of the positive effect incurred through the modality of telemedicine, and it serves as another facilitator to its adoption commensurate with the literature [70]. The treatment results were not always statistically different from treatment as usual; however, in every case, the treatment modality still resulted in a positive effect on symptoms, conditions, or behavior. This was an important finding because even if a treatment modality was not significantly better than treatment as usual, it might meet the digital preference of a patient.

Future research should expand some of these RCTs to help firmly establish telemedicine as an acceptable modality of care. This systematic literature review analyzed only 33 studies, but these studies focused on a wide range of specialties: tuberculosis, hypertension, alcohol consumption, mental health, HIV management, heart disease, smoking cessation, preventive medicine, stroke rehabilitation, nutrition, pain management, autism behavior management, diabetes management, Alzheimer disease, activity management, telerehabilitation for physical activity, and cancer recovery. Further research could expand on these specialties to identify where telemedicine is not an acceptable modality of care. After a family of systematic reviews was published, a review of these reviews summarized the effectiveness of telemedicine across all aspects of care.

This study has both practical and policy implications. Health care administrators should be confident in the investment of technology infrastructure to support the modality of telemedicine. The pandemic introduced transformational telehealth adoption, and restrictive regulations on modality were lifted [71]. Telemedicine is scalable and enables the web-based expansion of clinics without physically expanding the health care plant [71]. Providers should feel confident in the continued provision of telemedicine in their practice because it is rapidly becoming a preference for patients, even older adults, despite the technology gap [72,73]. Policy makers should encourage the modality of telemedicine because it increases access to care and saves patients the cost of travel and time off work [74].

### Limitations

This systematic literature review queried 4 research databases to control for sample bias. Additional research databases can also be queried. We only accepted published peer-reviewed literature to control for validity. Accepting gray literature could have better controlled for publication bias, but it may have introduced questionable internal and external validity. Our team has identified several instances of selection and sample bias. Our assessment was that their effect was small. However, it is possible that these instances could have presented significant challenges to both internal and external validity. To control for design bias, this systematic literature review used a previously published protocol. Other protocols could have been used. This review queried only 10 months of 2022 and only 33 articles were analyzed. Additional years and articles could have yielded more robust results.

### Conclusions

Telemedicine serves as an effective modality of care for a wide range of medical services, and its effectiveness has been demonstrated across all 6 domains of quality. These interventions have a positive effect on physical and mental health, engagement with the medical community, changed behavior, increased QoL, self-efficacy, and social support. This modality is patient-centered because it puts the patient’s schedule before the providers, saves time and mileage, avoids the stigma of care associated with some clinics, and patients often prefer it. The results of this systematic review should enable providers to adopt telemedicine as a standard option of care for patients. Studies with robust designs have shown telemedicine to be an effective modality of care, and it falls within the preference of many patients. Administrators should be confident in investing in technology to enable this modality of care. Policy makers should focus on removing the barriers to adoption.

## Appendix

Multimedia Appendix 1 Observation-to-theme conversion: intervention, results, medical outcomes.

Multimedia Appendix 2 Observation-to-theme conversion: patient satisfaction, facilitators, barriers, domains of quality.

Multimedia Appendix 3 Other observations incident to review.

## Abbreviations

JHNEBP: Johns Hopkins Nursing Evidence-Based Practice
PRISMA: Preferred Reporting Items for Systematic Reviews and Meta-Analyses
QOL: quality of life
RCT: randomized controlled trial

## Data Availability

Data from this study can be obtained by asking the lead author.

## References

[1] (2010). Telemedicine: opportunities and developments in Member States: report on the second global survey on eHealth. World Health Organization.

[2] (2011). mHealth: new horizons for health through mobile technologies: second global survey on eHealth. World Health Organization.

[3] Standage T (1998). The Victorian Internet: The Remarkable Story of the Telegraph and the Nineteenth Century's Online Pioneers.

[4] Vladzymyrskyy A, Jordanova M, Lievens F (2016). A Century of Telemedicine: Curatio Sine Distantia et Tempora.

[5] Betancourt JA, Rosenberg MA, Zevallos A, Brown JR, Mileski M (2020). The Impact of COVID-19 on Telemedicine Utilization Across Multiple Service Lines in the United States. Healthcare (Basel).

[6] Kruse C, Heinemann K (2022). Facilitators and barriers to the adoption of telemedicine during the first year of COVID-19: systematic review. J Med Internet Res.

[7] Kohn LT, Corrigan JM, Donaldson MS, Institute of Medicine (US) Committee on Quality of Health Care in America (2000). To Err is Human: Building a Safer Health System.

[8] Quinton JK, Ong MK, Sarkisian C, Casillas A, Vangala S, Kakani P, Han M (2022). The impact of telemedicine on quality of care for patients with diabetes after March 2020. J Gen Intern Med.

[9] Hernaez R, Kanwal F (2022). Leveraging telemedicine for quality assessment. Clin Liver Dis (Hoboken).

[10] Ostrowski-Delahanty SA, McNinch NL, Grossoehme DH, Aultman J, Spalding S, Wagoner C, Rush S (2022). Understanding drivers of telemedicine in pediatric medical care. Telemed J E Health (forthcoming).

[11] Love M, Hunter AK, Lam G, Muir LV, Lin HC (2022). Patient satisfaction and perceived quality of care with telemedicine in a pediatric gastroenterology clinic. Pediatr Rep.

[12] Fieux M, Duret S, Bawazeer N, Denoix L, Zaouche S, Tringali S (2020). Telemedicine for ENT: effect on quality of care during Covid-19 pandemic. Eur Ann Otorhinolaryngol Head Neck Dis.

[13] Shi Z, Mehrotra A, Gidengil CA, Poon SJ, Uscher-Pines L, Ray KN (2018). Quality of care for acute respiratory infections during direct-to-consumer telemedicine visits for adults. Health Aff (Millwood).

[14] Ferucci ED, Day GM, Choromanski TL, Freeman SL (2022). Outcomes and quality of care in rheumatoid arthritis with or without video telemedicine follow-up visits. Arthritis Care Res (Hoboken).

[15] Kruse CS, Betancourt JA, Madrid S, Lindsey CW, Wall V (2022). Leveraging mHealth and wearable sensors to manage alcohol use disorders: a systematic literature review. Healthcare (Basel).

[16] Alhajri N, Simsekler MC, Alfalasi B, Alhashmi M, Memon H, Housser E, Abdi AM, Balalaa N, Al Ali M, Almaashari R, Al Memari S, Al Hosani F, Al Zaabi Y, Almazrouei S, Alhashemi H (2022). Exploring quality differences in telemedicine between hospital outpatient departments and community clinics: cross-sectional study. JMIR Med Inform.

[17] Cui F, Ma Q, He X, Zhai Y, Zhao J, Chen B, Sun D, Shi J, Cao M, Wang Z (2020). Implementation and application of telemedicine in China: cross-sectional study. JMIR Mhealth Uhealth.

[18] Ye S, Anstey DE, Grauer A, Metser G, Moise N, Schwartz J, Kronish I, Abdalla M (2022). The impact of telemedicine visits on the controlling high blood pressure quality measure during the COVID-19 pandemic: retrospective cohort study. JMIR Form Res.

[19] Zhang W, Cheng B, Zhu W, Huang X, Shen C (2021). Effect of telemedicine on quality of care in patients with coexisting hypertension and diabetes: a systematic review and meta-analysis. Telemed J E Health.

[20] Page MJ, McKenzie JE, Bossuyt PM, Boutron I, Hoffmann TC, Mulrow CD, Shamseer L, Tetzlaff JM, Akl EA, Brennan SE, Chou R, Glanville J, Grimshaw JM, Hróbjartsson A, Lalu MM, Li T, Loder EW, Mayo-Wilson E, McDonald S, McGuinness LA, Stewart LA, Thomas J, Tricco AC, Welch VA, Whiting P, Moher D (2021). The PRISMA 2020 statement: an updated guideline for reporting systematic reviews. BMJ.

[21] Kruse CS (2019). Writing a systematic review for publication in a health-related degree program. JMIR Res Protoc.

[22] Newhouse R, Dearholt S, Poe S, Pugh LC, White K (2005). The Johns Hopkins nursing evidence-based practice rating scale. The Johns Hopkins Hospital.

[23] Pannucci CJ, Wilkins EG (2010). Identifying and avoiding bias in research. Plast Reconstr Surg.

[24] Shadish WR, Haddock CK, Cooper H, Hedges LV, Valnetine JC (2009). Combining estimates of effect size. The Handbook of Research Synthesis and Meta-Analysis. 2nd edition.

[25] Light RJ (1971). Measures of response agreement for qualitative data: some generalizations and alternatives. Psychol Bull.

[26] McHugh ML (2012). Interrater reliability: the kappa statistic. Biochem Med (Zagreb).

[27] Braun V, Clarke V (2006). Using thematic analysis in psychology. Qual Res Psychol.

[28] Bao Y, Wang C, Xu H, Lai Y, Yan Y, Ma Y, Yu T, Wu Y (2022). Effects of an mHealth intervention for pulmonary tuberculosis self-management based on the integrated theory of health behavior change: randomized controlled trial. JMIR Public Health Surveill.

[29] Bendtsen M, Åsberg K, McCambridge J (2022). Effectiveness of a digital intervention versus alcohol information for online help-seekers in Sweden: a randomised controlled trial. BMC Med.

[30] Bhandari B, Narasimhan P, Jayasuriya R, Vaidya A, Schutte AE (2022). Effectiveness and acceptability of a mobile phone text messaging intervention to improve blood pressure control (TEXT4BP) among patients with hypertension in Nepal: a feasibility randomised controlled trial. Glob Heart.

[31] Catuara-Solarz S, Skorulski B, Estella-Aguerri I, Avella-Garcia CB, Shepherd S, Stott E, Hemmings NR, Ruiz de Villa A, Schulze L, Dix S (2022). The efficacy of "Foundations," a digital mental health app to improve mental well-being during COVID-19: proof-of-principle randomized controlled trial. JMIR Mhealth Uhealth.

[32] Choi SK, Golinkoff J, Michna M, Connochie D, Bauermeister J (2022). Correlates of engagement within an online HIV prevention intervention for single young men who have sex with men: randomized controlled trial. JMIR Public Health Surveill.

[33] Dalli Peydró E, Sanz Sevilla N, Tuzón Segarra MT, Miró Palau V, Sánchez Torrijos J, Cosín Sales J (2022). A randomized controlled clinical trial of cardiac telerehabilitation with a prolonged mobile care monitoring strategy after an acute coronary syndrome. Clin Cardiol.

[34] do Amaral LM, Ronzani TM, Cruvinel E, Richter K, Oliveira Andrade RD, Lanzieri IO, de Macêdo ÂC, Leite IC (2022). Text messaging interventions to support smoking cessation among hospitalized patients in Brazil: a randomized comparative effectiveness clinical trial. BMC Res Notes.

[35] Fernandez ME, Savas LS, Atkinson JS, Ricks KB, Ibekwe LN, Jackson I, Castle PE, Jobe D, Vernon SW (2022). Evaluation of a 2-1-1 telephone navigation program to increase cancer control behaviors: results from a randomized controlled trial. Am J Health Promot.

[36] Guillaumier A, Spratt NJ, Pollack M, Baker A, Magin P, Turner A, Oldmeadow C, Collins C, Callister R, Levi C, Searles A, Deeming S, Clancy B, Bonevski B (2022). Evaluation of an online intervention for improving stroke survivors' health-related quality of life: a randomised controlled trial. PLoS Med.

[37] Gustafson DH, Kornfield R, Mares ML, Johnston DC, Cody OJ, Yang EF, Gustafson DH, Hwang J, Mahoney JE, Curtin JJ, Tahk A, Shah DV (2022). Effect of an eHealth intervention on older adults' quality of life and health-related outcomes: a randomized clinical trial. J Gen Intern Med.

[38] Huggins CE, Hanna L, Furness K, Silvers MA, Savva J, Frawley H, Croagh D, Cashin P, Low L, Bauer J, Truby H, Haines TP (2022). Effect of early and intensive telephone or electronic nutrition counselling delivered to people with upper gastrointestinal cancer on quality of life: a three-arm randomised controlled trial. Nutrients.

[39] Itoh N, Mishima H, Yoshida Y, Yoshida M, Oka H, Matsudaira K (2022). Evaluation of the effect of patient education and strengthening exercise therapy using a mobile messaging app on work productivity in Japanese patients with chronic low back pain: open-label, randomized, parallel-group trial. JMIR Mhealth Uhealth.

[40] Jamali AR, Alizadeh Zarei M, Sanjari MA, AkbarFahimi M, Saneii SH (2021). Randomized controlled trial of occupation performance coaching for families of children with autism spectrum disorder by means of telerehabilitation. Br J Occup Ther.

[41] Leong CM, Lee TI, Chien YM, Kuo LN, Kuo YF, Chen HY (2022). Social media-delivered patient education to enhance self-management and attitudes of patients with type 2 diabetes during the COVID-19 pandemic: randomized controlled trial. J Med Internet Res.

[42] María Gómez A, Cristina Henao D, León Vargas F, Mauricio Muñoz O, David Lucero O, García Jaramillo M, Aldea A, Martin C, Miguel Rodríguez Hortúa L, Patricia Rubio Reyes C, Alejandra Páez Hortúa M, Rondón M (2022). Efficacy of the mHealth application in patients with type 2 diabetes transitioning from inpatient to outpatient care: a randomized controlled clinical trial. Diabetes Res Clin Pract.

[43] Mathiasen K, Andersen TE, Lichtenstein MB, Ehlers LH, Riper H, Kleiboer A, Roessler KK (2022). The clinical effectiveness of blended cognitive behavioral therapy compared with face-to-face cognitive behavioral therapy for adult depression: randomized controlled noninferiority trial. J Med Internet Res.

[44] Molavynejad S, Miladinia M, Jahangiri M (2022). A randomized trial of comparing video telecare education vs. in-person education on dietary regimen compliance in patients with type 2 diabetes mellitus: a support for clinical telehealth Providers. BMC Endocr Disord.

[45] Morcillo-Muñoz Y, Sánchez-Guarnido AJ, Calzón-Fernández S, Baena-Parejo I (2022). Multimodal chronic pain therapy for adults via smartphone: randomized controlled clinical trial. J Med Internet Res.

[46] Muschol J, Heinrich M, Heiss C, Knapp G, Repp H, Schneider H, Thormann U, Uhlar J, Unzeitig K, Gissel C (2022). Assessing telemedicine efficiency in follow-up care with video consultations for patients in orthopedic and trauma surgery in Germany: randomized controlled trial. J Med Internet Res.

[47] Nagamitsu S, Kanie A, Sakashita K, Sakuta R, Okada A, Matsuura K, Ito M, Katayanagi A, Katayama T, Otani R, Kitajima T, Matsubara N, Inoue T, Tanaka C, Fujii C, Shigeyasu Y, Ishii R, Sakai S, Matsuoka M, Kakuma T, Yamashita Y, Horikoshi M (2022). Adolescent health promotion interventions using well-care visits and a smartphone cognitive behavioral therapy app: randomized controlled trial. JMIR Mhealth Uhealth.

[48] Ni Z, Wu B, Yang Q, Yan LL, Liu C, Shaw RJ (2022). An mHealth intervention to improve medication adherence and health outcomes among patients with coronary heart disease: randomized controlled trial. J Med Internet Res.

[49] Pires M, Shaha S, King C, Morrison J, Nahar T, Ahmed N, Jennings HM, Akter K, Haghparast-Bidgoli H, Khan AK, Costello A, Kuddus A, Azad K, Fottrell E (2022). Equity impact of participatory learning and action community mobilisation and mHealth interventions to prevent and control type 2 diabetes and intermediate hyperglycaemia in rural Bangladesh: analysis of a cluster randomised controlled trial. J Epidemiol Community Health.

[50] Pischke CR, Voelcker-Rehage C, Ratz T, Peters M, Buck C, Meyer J, von Holdt K, Lippke S (2022). Web-based versus print-based physical activity intervention for community-dwelling older adults: crossover randomized trial. JMIR Mhealth Uhealth.

[51] Roddy MK, Nelson LA, Greevy RA, Mayberry LS (2022). Changes in family involvement occasioned by FAMS mobile health intervention mediate changes in glycemic control over 12 months. J Behav Med.

[52] Şahin A, Agar A, Ertürk C (2022). The effect of telerehabilitation on early outcomes in patients undergoing primary total knee replacement: a prospective randomized study. J Surg Med.

[53] Sarker MH, Moriyama M, Rashid HU, Rahman MM, Chisti MJ, Das SK, Saha SK, Arifeen SE, Ahmed T, Faruque AS (2022). Chronic kidney disease awareness campaign and mobile health education to improve knowledge, quality of life, and motivation for a healthy lifestyle among patients with chronic kidney disease in Bangladesh: randomized controlled trial. J Med Internet Res.

[54] Seib C, Anderson D, McGuire A, Porter-Steele J, McDonald N, Balaam S, Sapkota D, McCarthy AL (2022). Improving health-related quality of life in women with breast, blood, and gynaecological Cancer with an eHealth-enabled 12-week lifestyle intervention: the women's wellness after Cancer program randomised controlled trial. BMC Cancer.

[55] Skvortsova A, Cohen Rodrigues T, de Buisonjé D, Kowatsch T, Santhanam P, Veldhuijzen DS, van Middendorp H, Evers A (2022). Increasing the effectiveness of a physical activity smartphone intervention with positive suggestions: randomized controlled trial. J Med Internet Res.

[56] Stephenson R, Sullivan SP, Mitchell JW, Johnson BA, Sullvian PS (2022). Efficacy of a telehealth delivered couples' HIV counseling and testing (CHTC) intervention to improve formation and adherence to safer sexual agreements among male couples in the US: results from a randomized control trial. AIDS Behav.

[57] Thesen T, Himle JA, Martinsen EW, Walseth LT, Thorup F, Gallefoss F, Jonsbu E (2022). Effectiveness of internet-based cognitive behavioral therapy with telephone support for noncardiac chest pain: randomized controlled trial. J Med Internet Res.

[58] Xia SF, Maitiniyazi G, Chen Y, Wu XY, Zhang Y, Zhang XY, Li ZY, Liu Y, Qiu YY, Wang J (2022). Web-based TangPlan and WeChat combination to support self-management for patients with type 2 diabetes: randomized controlled trial. JMIR Mhealth Uhealth.

[59] Zeng Y, Guo Y, Ho RT, Zhu M, Zeng C, Monroe-Wise A, Li Y, Qiao J, Zhang H, Cai W, Li L, Liu C (2022). Positive coping as a mediator of mobile health intervention effects on quality of life among people living with HIV: secondary analysis of the randomized controlled trial Run4Love. J Med Internet Res.

[60] Zhang L, Zhang X, Shen L, Zhu D, Ma S, Cong L (2022). Efficiency of electronic health record assessment of patient-reported outcomes after cancer immunotherapy: a randomized clinical trial. JAMA Netw Open.

[61] Hand DJ, Christen P, Kirielle N (2021). F*: an interpretable transformation of the F-measure. Mach Learn.

[62] Salgado JF (2018). Transforming the area under the normal curve (AUC) into Cohen’s d, Pearson’s rpb, odds-ratio, and natural log odds-ratio: two conversion tables. Eur J Psychol Appl Legal Context.

[63] Sharma AE, Rivadeneira NA, Barr-Walker J, Stern RJ, Johnson AK, Sarkar U (2018). Patient engagement in health care safety: an overview of mixed-quality evidence. Health Aff (Millwood).

[64] Coulter A (2012). Patient engagement--what works?. J Ambul Care Manage.

[65] Rollnick S, Miller WR, Butler CC (2008). Motivational Interviewing in Health Care: Helping Patients Change Behavior.

[66] Malik P, Patel K, Pinto C, Jaiswal R, Tirupathi R, Pillai S, Patel U (2022). Post-acute COVID-19 syndrome (PCS) and health-related quality of life (HRQoL)-A systematic review and meta-analysis. J Med Virol.

[67] Gilkey MB, Kong WY, Kennedy KL, Heisler-MacKinnon J, Faugno E, Gwinn B, Wu AC, Loughlin CE, Galbraith AA (2022). Leveraging telemedicine to reduce the financial burden of asthma care. J Allergy Clin Immunol Pract.

[68] Mink van der Molen DR, Bargon CA, Batenburg MC, van Stam LE, van Dam IE, Baas IO, Ernst MF, Maarse W, Sier M, Schoenmaeckers EJ, van Dalen T, Bijlsma RM, Doeksen A, van der Leij F, Young-Afat DA, Verkooijen HM, on behalf of UMBRELLA study group (2022). The impact of the COVID-19 pandemic on perceived access to health care and preferences for health care provision in individuals (being) treated for breast cancer. Breast Cancer Res Treat.

[69] Wegermann K, Wilder JM, Parish A, Niedzwiecki D, Gellad ZF, Muir AJ, Patel YA (2022). Racial and socioeconomic disparities in utilization of telehealth in patients with liver disease during COVID-19. Dig Dis Sci.

[70] Kruse CS, Beane A (2018). Health information technology continues to show positive effect on medical outcomes: systematic review. J Med Internet Res.

[71] Bakalar RS, Kiel JM, Kim GR, Ball MJ (2022). Telemedicine: its past, present and future. Healthcare Information Management Systems: Cases, Strategies, and Solutions.

[72] Alsabeeha NH, Atieh MA, Balakrishnan MS (2022). Older adults' satisfaction with telemedicine during the COVID-19 pandemic: a systematic review. Telemed J E Health (forthcoming).

[73] Mozes I, Mossinson D, Schilder H, Dvir D, Baron-Epel O, Heymann A (2022). Patients' preferences for telemedicine versus in-clinic consultation in primary care during the COVID-19 pandemic. BMC Prim Care.

[74] Greiwe J (2022). Telemedicine lessons learned during the COVID-19 pandemic. Curr Allergy Asthma Rep.

