# Supplementary material for: Analyzing the Effect of Telemedicine on Domains of Quality Through Facilitators and Barriers to Adoption: Systematic Review
Source: J Med Internet Res. 2023 Jan 5;25:e43601. doi: 10.2196/43601 (PMC9893735; doi:10.2196/43601)
Supplement: Multimedia Appendix 1 [file jmir_v25i1e43601_app1.docx]

**Appendix A:** Observation-to-theme conversion

| Authors | Experimental Intervention | Intervention themes | Results (compared to control group) | Result Theme | Medical Outcomes Reported | Outcome Theme |  |
| --- | --- | --- | --- | --- | --- | --- | --- |
| Bao et al [28] | mHealth (WeChat) for pulmonary Tuberculosis self-management | mHealth | increase in self-care management behaviors (self-efficacy) (*p*<0.001), increase in TB knowledge awareness, self-efficacy, social support, and degree of satisfaction with health education (*p*<0.001) compared with routine, in-person care in the clinic | Increase in self-efficacy | Increase in self-efficacy, TB knowledge, social support, and degree of satisfaction with health knowledge | Increase in self-efficacy |  |
|  |  |  |  | Improved medical engagement |  | Improved medical engagement |  |
|  |  |  |  | Increase in social support |  | Increase in social support |  |
|  |  |  |  |  |  |  |  |
|  |  |  |  |  |  |  |  |
|  |  |  |  |  |  |  |  |
| Bendtsen et al [29] | mHealth app for self-reporting of alcohol consumption | mHealth | Decreased drinking (*p*=0.033) more than the control group (traditional counseling). Changed behavior. | Changed behavior | Decreased drinking and changed behavior | Changed behavior |  |
|  |  |  |  |  |  |  |  |
|  |  |  |  |  |  |  |  |
|  |  |  |  |  |  |  |  |
|  |  |  |  |  |  |  |  |
|  |  |  |  |  |  |  |  |
|  |  |  |  |  |  |  |  |
| Bhandari et al [30] | mHealth SMS (TEXT4BP) to improve blood pressure | mHealth | Decreased diastolic BP (*p*<0.001), systolic (*p*<0.001) and increase in therapy compliance (*p*<0.001), medication adherence (*p*<0.023), and knowledge (*p*<0.013) over the control (usual treatment). | Increase in physical health | Decreased blood pressure, increased therapy compliance, increased medication adherence, increase in hypertension knowledge | Increase in physical health |  |
|  |  |  |  | Increase in physical health |  | Improved medical engagement |  |
|  |  |  |  | Improved medical engagement |  | Improved medical engagement |  |
|  |  |  |  | Improved medical engagement |  | Improved medical engagement |  |
|  |  |  |  | Improved medical engagement |  |  |  |
|  |  |  |  | Improved medical engagement |  |  |  |
| Catuara-Solarz et al [31] | mHealth app for mental health | mHealth | Decrease in anxiety (*p*=.04), resilience (*p*<0.001), sleep (*p*=0.01), mental well-being (*p*=0.02), and stress (*p*=0.2) relative to the control group | Increase in mental health | Decreased anxiety, increased resilience, increased sleep, increased mental well-being, and decreased stress | Increase in mental health |  |
|  |  |  |  | Increase in mental health |  | Increase in mental health |  |
|  |  |  |  | Increased QoL |  | Increased QoL |  |
|  |  |  |  | Increase in mental health |  | Increase in mental health |  |
|  |  |  |  | Increase in mental health |  | Increase in mental health |  |
| Choi et al [32] | Digital HIV intervention (myDex) | eHealth | Increase in education (*p*=0.003), decrease in loneliness (*p*=0.004), lower experience of online discrimination (*p*=0.007), but no difference in behavior at 90 day follow-up. Changed dangerous sexual behavior | Improved medical engagement | Increase in education, decrease in loneliness, decrease in online discrimination, decreased dangerous sexual behavior but not significantly | Improved medical engagement |  |
|  |  |  |  | Increase in mental health |  | Increase in mental health |  |
|  |  |  |  | Increase in social support |  | Increase in social support |  |
|  |  |  |  | Changed behavior |  | Changed behavior |  |
| Dalli et al [33] | cardiac telerehabilitation | Telehealth | Increased mean VO2max (*p*<0.004), decreased apoB/apoA=I ration (*p*=0.017), increase in physical activity, return to work was reduced with intervention | Increase in physical health | Increased VO2max, decrease in apoB/apoA-I, increase in physical activity | Increase in physical health |  |
|  |  |  |  | Increase in physical health |  | Increase in physical health |  |
|  |  |  |  | Changed behavior |  | Changed behavior |  |
| do Amaral et al [34] | mHealth SMS for smoking cessation | mHealth | Costs were lower (*p*<0.001), continuous abstinence reported by both groups. | Reduced costs | Decreased smoking (continuous abstinence) | Changed behavior |  |
|  |  |  |  | Changed behavior |  |  |  |
| Fernandez et al [35] | telephone navigation service | Telephone | Intervention resulted in greater completion of needed service (*p*=0.042), Pap test (*p*=0.023), and smoking cessation (*p*=0.044). Other areas were improved, but not statistically significant | Improved medical engagement | Greater completion rates, more Pap tests, greater smoking cessation, completed mammograms, colorectal cancer screening and HPV vaccinations | Improved medical engagement |  |
|  |  |  |  | Increase in self-efficacy |  | Increase in self-efficacy |  |
|  |  |  |  | Increase in self-efficacy |  | Increase in self-efficacy |  |
| Guillaumier et al [36] | eHealth app (Prevent 2nd Stroke, P2S) | eHealth | Quality of life significantly higher for intervention (*p*=0.020), reported no problems with personal care (*p*=0.0359) and usual activities (*p*=0.0256). | Increased QoL | Increased quality of life, increased self-efficacy, increased usual activities | Increased QoL |  |
|  |  |  |  | Increase in self-efficacy |  | Increase in self-efficacy |  |
|  |  |  |  | Improved medical engagement |  | Improved medical engagement |  |
| Gustafson et al [37] | eHealth app (ElderTree) | eHealth | Improved depression (OR=-0.20, *p*=.034)and overall mental health quality of life (OR=0.32, *p*=.007) more than the control group | Increase in mental health | Decreased depression, increased mental health, increased quality of life | Increase in mental health |  |
|  |  |  |  | Increased QoL |  | Increased QoL |  |
| Huggins et al [38] | Telephone or electronic nutrition counseling | Telephone | No statistical difference in quality adjusted life years than treatment as usual | Increased QALYs | Increased QALYs | Increased QALYs |  |
| Itoh et al [39] | mHealth app for patient education and strengthening exercise therapy | mHealth | Intervention group reported less back pain (*p*=0.04), higher quality of life (*p*=0.03), and less fear of movement at week 12 (*p*=0.04) | Increase in physical health | Less back pain, improved quality of life, less fear of movement | Increase in physical health |  |
|  |  |  |  | Increased QoL |  | Increased QoL |  |
|  |  |  |  | Increase in mental health |  | Increase in mental health |  |
| Jamali et al [40] | WhatsApp coaching intervention | mHealth | Intervention group shows greater improvement in occupational performance, specified goals, and behavioral problems. | Increase in physical health | Improved occupational performance, improved specified goals, improved behavioral problems | Increase in physical health |  |
|  |  |  |  | Improved medical engagement |  | Improved medical engagement |  |
|  |  |  |  | Increased QoL |  | Increased QoL |  |
| Leong et al [41] | Social media-delivered patient education | mHealth | Change in HbA1C not significant, intervention group showed positive improvements in attitudes (*p*<0.001) and self-care activities (*p*=0.03). Low health literacy contributed to baseline knowledge (*p*=0.01) | Increase in physical health | Improve HbA1c, Increase in self-efficacy, increase in attitude | Increase in physical health |  |
|  |  |  |  | Increase in self-efficacy |  | Increase in self-efficacy |  |
|  |  |  |  | Increased QoL |  | Increased QoL |  |
| María Gómez et al [42] | mHealth app (DM2) | mHealth | Lower HbA1c levels in intervention group, decreased incidence of hypoglycemia 3.00 mmol/L and severe hypoglycemia | Increase in physical health | Decreased HbA1c, decreased incidence of hypoglycemia and severe hypoglycemia | Increase in physical health |  |
|  |  |  |  | Increase in physical health |  | Increase in physical health |  |
|  |  |  |  | Increase in physical health |  | Increase in physical health |  |
| Mathiasen et al [43] | Internet-based CBT | eHealth | Therapy compliance not as statistically high as TAU, decreases in depression not as statistically much as TAU | Improved medical engagement | Maintained therapy compliance, decreased symptoms of depression comparable with treatment as usual | Improved medical engagement |  |
|  |  |  |  | Increase in mental health |  | Increase in mental health |  |
| Molavynejad et al [44] | Video telecare education | eHealth | mean changes of patients’ weight, glycemic parameters, and Lipid profiles decreased more in the two educational groups than the control group | Increase in physical health | Lost weight, lower glycemic parameters, lower lipid profiles | Increase in physical health |  |
|  |  |  |  | Increase in physical health |  | Increase in physical health |  |
|  |  |  |  | Increase in physical health |  | Increase in physical health |  |
| Morcillo-Muñoz et al [45] | web-based psychosocial chronic pain therapy | eHealth | Intervention group showed lower catastrophizing (*p*<0.001), less helplessness (*p*=0.002), and improved rumination (*p*<0.001), acceptance (*p*=0.001), and quality of life (*p*=0.002) over the control. No significant changes reported in magnification and satisfaction with health. | Increase in mental health | Improved catastrophizing, helplessness, rumination, acceptance, and quality of life. Improvements were also noted in magnification and satisfaction, but these were not statistically significant. | Increase in mental health |  |
|  |  |  |  | Increase in mental health |  | Increase in mental health |  |
|  |  |  |  | Increase in mental health |  | Increase in mental health |  |
|  |  |  |  | Increase in mental health |  | Increase in mental health |  |
|  |  |  |  | Increased QoL |  | Increased QoL |  |
| Muschol et al [46] | Telephone video consultations | Telephone | The participants from the intervention group reported higher satisfaction, but it was not statistically significant (*p*=0.69) | Improved medical engagement | Improved satisfaction | Improved medical engagement |  |
| Nagamitsu et al[47] | iCBT | eHealth | Intervention group reported reduced scores for depressive symptoms and suicide ideation, increase in health promotion, and improved self-monitoring skills to reduce depressive symptoms | Increase in mental health | Improved depression, less suicide ideation, more self-efficacy & health promotion | Increase in mental health |  |
|  |  |  |  | Increase in mental health |  | Increase in mental health |  |
|  |  |  |  | Increase in physical health |  | Increase in physical health |  |
|  |  |  |  | Increase in self-efficacy |  | Increase in self-efficacy |  |
| Ni et al [48] | mHealth (WeChat and Message Express) to improve medication adherence | mHealth | Intervention group showed increase in medication adherence and decrease in systolic blood pressure | Improved medical engagement | Increased medication adherence and decrease in blood pressure | Improved medical engagement |  |
|  |  |  |  | Increase in physical health |  | Increase in physical health |  |
| Pires et al [49] | mHealth app for diabetes management | mHealth | Intervention group decreased the prevalence of T2DM and intermediate hyperglycemia. | Increase in physical health | Improved symptoms of type 2 diabetes mellitus | Increase in physical health |  |
|  |  |  |  | Increase in physical health |  |  |  |
| Pischke et al [50] | eHealth physical activity intervention | eHealth | Intervention showed increased MVPA | Changed behavior | Increased activity | Changed behavior |  |
| Roddy et al [51] | mHealth (FAMS) for glycemic control | mHealth | Family involvement helped decrease HbA1c | Increase in physical health | Decreased HbA1c | Increase in physical health |  |
| Sahin et al [52] | telerehabilitation for patient with knee replacements | Telehealth | Intervention group demonstrated improvements in movement on the Barthal Index (BI) (*p*<0.001) | Increase in physical health | Improved physical function of knee | Increase in physical health |  |
| Sarker et al [53] | mHealth disease education | mHealth | Intervention group demonstrated lower diastolic BP, lower BMI, and lower salt intake | Increase in physical health | Improved diet, decreased BMI, reduced BP | Changed behavior |  |
|  |  |  |  | Increase in physical health |  | Increase in physical health |  |
|  |  |  |  | Changed behavior |  | Increase in physical health |  |
| Seib et al [54] | eHealth cancer intervention | eHealth | Intervention group demonstrated improved general health, bodily pain, vitality, and global physical and mental health scores | Increase in physical health | Improved physical and mental health, decreased pain, increased vitality | Increase in physical health |  |
|  |  |  |  | Increase in physical health |  | Increase in physical health |  |
|  |  |  |  | Increased QoL |  | Increased QoL |  |
|  |  |  |  | Increase in mental health |  | Increase in mental health |  |
| Skvortsova et al[55] | mHealth physical activity intervention | mHealth | Intervention participants increased daily step count (*p*<0.001) | Increase in physical health | Increased activity | Increase in physical health |  |
|  |  |  |  | Changed behavior |  | Changed behavior |  |
| Stephenson et al [56] | Telehealth couples counseling and testing (CHTC) | Telehealth | Couples in the intervention group reported safer sexual agreements (*p*=0.007), lower odds of discordant relationships (*p*=0.048), lower odds of breaking their sexual agreement (*p*=0.000) | Changed behavior | Decreased interpersonal problems | Increased QoL |  |
|  |  |  |  | Increased QoL |  | Changed behavior |  |
|  |  |  |  | Changed behavior |  |  |  |
| Thesen et al [57] | iCBT | eHealth | Intervention group demonstrated improvements in cardiac anxiety (*p*=0.004), and a non-significant improvement in fear of bodily sensations (*p*=0.07). Improvement in health-related QoL (*p*=0.004), increase in physical activity (*p*<0.001), improvement in depression (*p*=0.03) | Increase in physical health | Improvement in cardiac anxiety, increased health-related QoL, increased physical activity, improved depression | Increase in physical health |  |
|  |  |  |  | Increase in mental health |  | Increase in mental health |  |
|  |  |  |  | Increased QoL |  | Increased QoL |  |
| Xia et al [58] | WeChat + T2DM (TangPlan) to support pts with Type 2 Diabetes | mHealth | The intervention group demonstrated improved fasting blood glucose, FBG (*p*=0.048), HbA1c (*p*<0.001), body weight (*p*=0.006), systolic BP (*p*=0.005), diastolic BP (*p*=0.03), serum low-density lipoprotein cholesterol (*p*=0.006), and cholesterol mean (*p*=0.02). | Increase in physical health | Improved FBG, HbA1c, weight, systolic and diastolic BP, serum low-density lipoprotein cholesterol, and cholesterol mean | Increase in physical health |  |
|  |  |  |  | Increase in physical health |  | Increase in physical health |  |
|  |  |  |  | Changed behavior |  | Changed behavior |  |
|  |  |  |  | Increase in physical health |  | Increase in physical health |  |
|  |  |  |  | Increase in physical health |  | Increase in physical health |  |
|  |  |  |  | Increase in physical health |  | Increase in physical health |  |
|  |  |  |  | Increase in physical health |  | Increase in physical health |  |
| Zeng et al [59] | mHealth WeChat app (Run4Love) | mHealth | increased quality of life through positive coping (*p*=0.006) over control | Increased QoL | Increased quality of life | Increased QoL |  |
|  |  |  |  | Changed behavior |  | Changed behavior |  |
| Zhang et al [60] | mHealth questionnaires with follow-up | mHealth | Intervention group showed fewer immune-related adverse events (irAEs) (*p*=0.01), fewer ED visits (*p*=0.01), lower rate of treatment discontinuation (*p*=0.02), higher quality of life (*p*=0.001), and less time implementing follow-up (*p*=0.28) | Fewer irAEs | fewer irAEs, fewer ED visits, better treatment engagement, higher QoL, better follow-up | Fewer irAEs |  |
|  |  |  |  | Changed behavior |  | Changed behavior |  |
|  |  |  |  | Improved medical engagement |  | Improved medical engagement |  |
|  |  |  |  | Increased QoL |  | Increased QoL |  |
|  |  |  |  | Improved medical engagement |  | Improved medical engagement |  |
