# Supplementary material for: Analyzing the Effect of Telemedicine on Domains of Quality Through Facilitators and Barriers to Adoption: Systematic Review
Source: J Med Internet Res. 2023 Jan 5;25:e43601. doi: 10.2196/43601 (PMC9893735; doi:10.2196/43601)
Supplement: Multimedia Appendix 2 [file jmir_v25i1e43601_app2.docx]

**Appendix B:** Observation-to-theme conversion

| Authors | Patient Satisfaction | Satisfaction Theme | Facilitators to adoption | Facilitator Theme | Barriers to adoption | Barrier Theme | Domain of Quality | Domain of Quality Theme |  |
| --- | --- | --- | --- | --- | --- | --- | --- | --- | --- |
| Bao et al [28] | Patients highly satisfied with health knowledge provided by app | Satisfied | Pts value technology, convenience, savings in time and mileage driven, meets a digital preference, education at own pace, Effective | Patients value technology | Staff training, may not be preferred | Staff training | Safe, Timely, Effective, Efficient, and Patient-centered | Safe - Avoiding harm |  |
|  |  |  |  | Convenience |  | May not be preferred modality |  | Timely - Reduce wait times |  |
|  |  |  |  | Savings in time and mileage |  |  |  | Effective - Evidence based |  |
|  |  |  |  | Meets a digital preference |  |  |  | Patient-centered - Respect autonomy |  |
|  |  |  |  | Education at own pace |  |  |  |  |  |
|  |  |  |  | Effective |  |  |  |  |  |
| Bendtsen et al [29] | Positive effect on patient experience | Satisfied | Pts value technology, convenience, savings in time and mileage driven, meets a digital preference, education at own pace, avoids stigma of alcoholism by treating outside the clinic, Effective | Patients value technology | Cost to acquire equipment, staff training, may not be preferred modality | Cost | Timely, effective, safe, Efficient, and patient-centered | Safe - Avoiding harm |  |
|  |  |  |  | Convenience |  | Staff training |  | Timely - Reduce wait times |  |
|  |  |  |  | Savings in time and mileage |  | May not be preferred modality |  | Effective - Evidence based |  |
|  |  |  |  | Meets a digital preference |  |  |  | Efficient - Lean |  |
|  |  |  |  | Meets a digital preference |  |  |  | Patient-centered - Respect autonomy |  |
|  |  |  |  | Avoids stigma |  |  |  |  |  |
|  |  |  |  | Effective |  |  |  |  |  |
| Bhandari et al [30] | Participants expressed a high rate of acceptability and desire to continue the intervention. Users reported the messages were useful, culturally appropriate, and age appropriate. | Satisfied | Pts value technology, convenience, savings in time and mileage driven, meets a digital preference, education at own pace, Effective | Patients value technology | Cost to acquire equipment, staff training, may not be preferred modality | Cost | Timely, Safe, Effective, Efficient, and Patient-centered | Safe - Avoiding harm |  |
|  |  |  |  | Convenience |  | Staff training |  | Timely - Reduce wait times |  |
|  |  |  |  | Savings in time and mileage |  | May not be preferred modality |  | Effective - Evidence based |  |
|  |  |  |  | Meets a digital preference |  |  |  | Efficient - Lean |  |
|  |  |  |  | Education at own pace |  |  |  | Patient-centered - Respect autonomy |  |
|  |  |  |  | Effective |  |  |  |  |  |
| Catuara-Solarz et al [31] | High app engagement indicates high satisfaction | Satisfied | Effective, pts value technology, convenient, savings in time and mileage driven, meets a digital preference | Effective | Cost to acquire equipment, staff training | Cost | Timely, Safe, Effective, Efficient, and Patient-centered | Safe - Avoiding harm |  |
|  |  |  |  | Patients value technology |  | Staff training |  | Timely - Reduce wait times |  |
|  |  |  |  | Savings in time and mileage |  |  |  | Effective - Evidence based |  |
|  |  |  |  | Meets a digital preference |  |  |  | Efficient - Lean |  |
|  |  |  |  |  |  |  |  | Patient-centered - Respect autonomy |  |
| Choi et al [32] | High app engagement indicates high satisfaction | Satisfied | Effective, convenient, Intervention meets digital preference, no HIV stigma | Effective | Staff training, low reimbursement, cost of app, may not be preferred modality | Staff training | Timely, Safe, effective, Patient-centered, and Efficient, Equitable | Safe - Avoiding harm |  |
|  |  |  |  | Convenience |  | Low reimbursement |  | Timely - Reduce wait times |  |
|  |  |  |  | Meets a digital preference |  | Cost |  | Efficient - Lean |  |
|  |  |  |  | Avoids stigma |  | May not be preferred modality |  | Effective - Evidence based |  |
|  |  |  |  |  |  |  |  | Patient-centered - Respect autonomy |  |
|  |  |  |  |  |  |  |  | Equitable - No variance based on personal characteristics |  |
| Dalli et al [33] | Positive patient experience with telerehabilitation | Satisfied | Effective, pts value technology, convenient, savings in time and mileage driven, meets a digital preference | Effective | Cost to acquire equipment, staff training, may not be preferred modality | Cost | Timely, Safe, Effective, Efficient, and Patient-centered | Safe - Avoiding harm |  |
|  |  |  |  | Patients value technology |  | Staff training |  | Timely - Reduce wait times |  |
|  |  |  |  | Convenience |  | May not be preferred modality |  | Effective - Evidence based |  |
|  |  |  |  | Savings in time and mileage |  |  |  | Efficient - Lean |  |
|  |  |  |  | Meets a digital preference |  |  |  | Patient-centered - Respect autonomy |  |
| do Amaral et al [34] | Positive patient experience with SMS messages | Satisfied | Effective, pts value technology, convenient, savings in time and mileage driven, meets a digital preference | Effective | Cost to acquire equiment, staff training, may not be preferred modality | Cost | Timely, Safe, Effective, Efficient, and Patient-centered | Safe - Avoiding harm |  |
|  |  |  |  | Patients value technology |  | Staff training |  | Timely - Reduce wait times |  |
|  |  |  |  | Convenience |  | May not be preferred modality |  | Effective - Evidence based |  |
|  |  |  |  | Savings in time and mileage |  |  |  | Efficient - Lean |  |
|  |  |  |  | Meets a digital preference |  |  |  | Patient-centered - Respect autonomy |  |
| Fernandez et al [35] | positive experience | Satisfied | Effective, pts value the personal navigation, convenient | Effective | Cost to hire navigators, low reimbursement | Cost | Timely, Safe, Effective, Efficient, Equitable, and Patient-centered | Safe - Avoiding harm |  |
|  |  |  |  | Patients value personal guidance |  | Low reimbursement |  | Timely - Reduce wait times |  |
|  |  |  |  | Convenience |  |  |  | Effective - Evidence based |  |
|  |  |  |  |  |  |  |  | Efficient - Lean |  |
|  |  |  |  |  |  |  |  | Patient-centered - Respect autonomy |  |
| Guillaumier et al [36] | positive experience | Satisfied | Effective, pts value technology, convenient, savings in time and mileage driven, meets a digital preference | Effective | Cost to acquire equipment, staff training, may not be preferred modality | Cost | Timely, Effective, Efficient, Safe, and Patient-centered | Safe - Avoiding harm |  |
|  |  |  |  | Patients value technology |  | Staff training |  | Timely - Reduce wait times |  |
|  |  |  |  | Convenience |  | May not be preferred modality |  | Effective - Evidence based |  |
|  |  |  |  | Savings in time and mileage |  |  |  | Efficient - Lean |  |
|  |  |  |  | Meets a digital preference |  |  |  | Patient-centered - Respect autonomy |  |
| Gustafson et al [37] | Not reported | Not reported | Effective, pts value technology, convenient, savings in time and mileage driven, meets a digital preference | Effective | May not be preferred modality, staff training, low reimbursement, cost of app | May not be preferred modality | Timely, Effective, Efficient, Safe, and Patient-centered | Safe - Avoiding harm |  |
|  |  |  |  | Patients value technology |  | Staff training |  | Timely - Reduce wait times |  |
|  |  |  |  | Convenience |  | Low reimbursement |  | Effective - Evidence based |  |
|  |  |  |  | Savings in time and mileage |  | Cost |  | Efficient - Lean |  |
|  |  |  |  | Meets a digital preference |  |  |  | Patient-centered - Respect autonomy |  |
| Huggins et al [38] | Low acceptance | Not satisfied | Effective, Pts value technology, convenience, savings in time and mileage driven, meets a digital preference, education at own pace | Effective | May not be preferred modality, staff training | May not be preferred modality | Timely, Effective, Efficient, Safe, Equitable, and Patient-centered | Safe - Avoiding harm |  |
|  |  |  |  | Patients value technology |  | Staff training |  | Timely - Reduce wait times |  |
|  |  |  |  | Convenience |  |  |  | Effective - Evidence based |  |
|  |  |  |  | Savings in time and mileage |  |  |  | Efficient - Lean |  |
|  |  |  |  | Meets a digital preference |  |  |  | Equitable - No variance based on personal characteristics |  |
|  |  |  |  | Education at own pace |  |  |  | Patient-centered - Respect autonomy |  |
| Itoh et al [39] | Well received, and it improved work productivity | Satisfied | Effective, Pts value technology, convenience, savings in time and mileage driven, meets a digital preference, education at own pace | Effective | May not be preferred modality, staff training, cost of app | May not be preferred modality | Timely, Effective, Efficient, Safe, and Patient-centered | Safe - Avoiding harm |  |
|  |  |  |  | Patients value technology |  | Staff training |  | Timely - Reduce wait times |  |
|  |  |  |  | Convenience |  | Cost |  | Effective - Evidence based |  |
|  |  |  |  | Savings in time and mileage |  |  |  | Efficient - Lean |  |
|  |  |  |  | Meets a digital preference |  |  |  | Patient-centered - Respect autonomy |  |
|  |  |  |  | Education at own pace |  |  |  |  |  |
| Jamali et al [40] | Well received | Satisfied | Effective, pts value technology, convenient, savings in time and mileage driven, meets a digital preference | Effective | May not be preferred modality, staff training, cost of app | May not be preferred modality | Timely, Effective, Efficient, Safe, and Patient-centered | Safe - Avoiding harm |  |
|  |  |  |  | Patients value technology |  | Staff training |  | Timely - Reduce wait times |  |
|  |  |  |  | Convenience |  | Cost |  | Effective - Evidence based |  |
|  |  |  |  | Savings in time and mileage |  |  |  | Efficient - Lean |  |
|  |  |  |  | Meets a digital preference |  |  |  | Patient-centered - Respect autonomy |  |
| Leong et al [41] | Not reported | Not reported | Effective, pts value technology, convenient, savings in time and mileage driven, meets a digital preference | Effective | May not be preferred modality, staff training, cost of app | May not be preferred modality | Timely, Effective, Efficient, Safe, and Patient-centered | Safe - Avoiding harm |  |
|  |  |  |  | Patients value technology |  | Staff training |  | Timely - Reduce wait times |  |
|  |  |  |  | Convenience |  | Cost |  | Effective - Evidence based |  |
|  |  |  |  | Savings in time and mileage |  |  |  | Efficient - Lean |  |
|  |  |  |  | Meets a digital preference |  |  |  | Patient-centered - Respect autonomy |  |
| María Gómez et al [42] | High satisfaction in the intervention group | Satisfied | Effective, pts value technology, convenient, savings in time and mileage driven, meets a digital preference | Effective | May not be preferred modality, staff training, cost of app | May not be preferred modality | Timely, Effective, Efficient, Safe, and Patient-centered | Safe - Avoiding harm |  |
|  |  |  |  | Patients value technology |  | Staff training |  | Timely - Reduce wait times |  |
|  |  |  |  | Convenience |  | Cost |  | Effective - Evidence based |  |
|  |  |  |  | Savings in time and mileage |  |  |  | Efficient - Lean |  |
|  |  |  |  | Meets a digital preference |  |  |  | Patient-centered - Respect autonomy |  |
| Mathiasen et al [43] | High satisfaction in the intervention group | Satisfied | Effective, Pts value technology, convenience, savings in time and mileage driven, meets a digital preference | Effective | May not be preferred modality, staff training | May not be preferred modality | Timely, Effective, Efficient, Safe, and Patient-centered | Safe - Avoiding harm |  |
|  |  |  |  | Patients value technology |  | Staff training |  | Timely - Reduce wait times |  |
|  |  |  |  | Convenience |  |  |  | Effective - Evidence based |  |
|  |  |  |  | Savings in time and mileage |  |  |  | Efficient - Lean |  |
|  |  |  |  | Meets a digital preference |  |  |  | Patient-centered - Respect autonomy |  |
| Molavynejad et al [44] | Patients were satisfied with the video education | Satisfied | Effective, Pts value technology, convenience, savings in time and mileage driven, meets a digital preference, education at own pace | Effective | May not be preferred modality, staff training | May not be preferred modality | Timely, Effective, Efficient, Safe, and Patient-centered | Safe - Avoiding harm |  |
|  |  |  |  | Patients value technology |  | Staff training |  | Timely - Reduce wait times |  |
|  |  |  |  | Convenience |  |  |  | Effective - Evidence based |  |
|  |  |  |  | Savings in time and mileage |  |  |  | Efficient - Lean |  |
|  |  |  |  | Meets a digital preference |  |  |  | Patient-centered - Respect autonomy |  |
|  |  |  |  | Education at own pace |  |  |  |  |  |
| Morcillo-Muñoz et al [45] | large dropout rate indicates lack of acceptance and satisfaction | Not satisfied | Effective, pts value technology, convenient, savings in time and mileage driven, meets a digital preference | Effective | Cost to acquire equipment, staff training, may not be preferred modality | Cost | Timely, Effective, Efficient, Safe, and Patient-centered | Safe - Avoiding harm |  |
|  |  |  |  | Patients value technology |  | Staff training |  | Timely - Reduce wait times |  |
|  |  |  |  | Convenience |  | May not be preferred modality |  | Effective - Evidence based |  |
|  |  |  |  | Savings in time and mileage |  |  |  | Efficient - Lean |  |
|  |  |  |  | Meets a digital preference |  |  |  | Patient-centered - Respect autonomy |  |
| Muschol et al [46] | Positive satisfaction | Satisfied | Effective, pts value technology, convenient, savings in time and mileage driven, meets a digital preference | Effective | Cost to acquire equipment, staff training, may not be preferred modality | Cost | Timely, Effective, Efficient, Safe, Equitable, and Patient-centered | Safe - Avoiding harm |  |
|  |  |  |  | Patients value technology |  | Staff training |  | Timely - Reduce wait times |  |
|  |  |  |  | Convenience |  | May not be preferred modality |  | Effective - Evidence based |  |
|  |  |  |  | Savings in time and mileage |  |  |  | Efficient - Lean |  |
|  |  |  |  | Meets a digital preference |  |  |  | Patient-centered - Respect autonomy |  |
| Nagamitsu et al [47] | Positive satisfaction | Satisfied | Effective, pts value technology, convenient, savings in time and mileage driven, meets a digital preference | Effective | Cost to acquire equipment, staff training, may not be preferred modality | Cost | Timely, Effective, Efficient, Safe, and Patient-centered | Safe - Avoiding harm |  |
|  |  |  |  | Patients value technology |  | Staff training |  | Timely - Reduce wait times |  |
|  |  |  |  | Convenience |  | May not be preferred modality |  | Effective - Evidence based |  |
|  |  |  |  | Savings in time and mileage |  |  |  | Efficient - Lean |  |
|  |  |  |  | Meets a digital preference |  |  |  | Patient-centered - Respect autonomy |  |
| Ni et al [48] | Positive satisfaction | Satisfied | Effective, pts value technology, convenient, savings in time and mileage driven, meets a digital preference | Effective | Cost to acquire equipment, staff training, may not be preferred modality | Cost | Timely, Effective, Efficient, Safe, and Patient-centered | Safe - Avoiding harm |  |
|  |  |  |  | Patients value technology |  | Staff training |  | Timely - Reduce wait times |  |
|  |  |  |  | Convenience |  | May not be preferred modality |  | Effective - Evidence based |  |
|  |  |  |  | Savings in time and mileage |  |  |  | Efficient - Lean |  |
|  |  |  |  | Meets a digital preference |  |  |  | Patient-centered - Respect autonomy |  |
| Pires et al [49] | Not reported | Not reported | Effective, pts value technology, convenient, savings in time and mileage driven, meets a digital preference | Effective | Cost to acquire equipment, staff training, may not be preferred modality | Cost | Timely, Effective, Efficient, Safe, and Patient-centered | Safe - Avoiding harm |  |
|  |  |  |  | Patients value technology |  | Staff training |  | Timely - Reduce wait times |  |
|  |  |  |  | Convenience |  | May not be preferred modality |  | Effective - Evidence based |  |
|  |  |  |  | Savings in time and mileage |  |  |  | Efficient - Lean |  |
|  |  |  |  | Meets a digital preference |  |  |  | Patient-centered - Respect autonomy |  |
| Pischke et al [50] | Positive satisfaction | Satisfied | Effective, pts value technology, convenient, savings in time and mileage driven, meets a digital preference | Effective | Cost to acquire equipment, staff training, may not be preferred modality | Cost | Timely, Effective, Efficient, Safe, and Patient-centered | Safe - Avoiding harm |  |
|  |  |  |  | Patients value technology |  | Staff training |  | Timely - Reduce wait times |  |
|  |  |  |  | Convenience |  | May not be preferred modality |  | Effective - Evidence based |  |
|  |  |  |  | Savings in time and mileage |  |  |  | Efficient - Lean |  |
|  |  |  |  | Meets a digital preference |  |  |  | Patient-centered - Respect autonomy |  |
| Roddy et al [51] | Not reported | Not reported | Effective, pts value technology, convenient, savings in time and mileage driven, meets a digital preference | Effective | Cost to acquire equipment, staff training, may not be preferred modality | Cost | Timely, Effective, Efficient, Safe, and Patient-centered | Safe - Avoiding harm |  |
|  |  |  |  | Patients value technology |  | Staff training |  | Timely - Reduce wait times |  |
|  |  |  |  | Convenience |  | May not be preferred modality |  | Effective - Evidence based |  |
|  |  |  |  | Savings in time and mileage |  |  |  | Efficient - Lean |  |
|  |  |  |  | Meets a digital preference |  |  |  | Patient-centered - Respect autonomy |  |
| Sahin et al [52] | Not reported | Not reported | Effective, pts value technology, convenient, savings in time and mileage driven, meets a digital preference, education at own pace | Effective | Cost to acquire equipment, staff training, may not be preferred modality | Cost | Timely, Effective, Efficient, Safe, and Patient-centered | Safe - Avoiding harm |  |
|  |  |  |  | Patients value technology |  | Staff training |  | Timely - Reduce wait times |  |
|  |  |  |  | Convenience |  | May not be preferred modality |  | Effective - Evidence based |  |
|  |  |  |  | Savings in time and mileage |  |  |  | Efficient - Lean |  |
|  |  |  |  | Meets a digital preference |  |  |  | Patient-centered - Respect autonomy |  |
|  |  |  |  | Education at own pace |  |  |  |  |  |
| Sarker et al [53] | Not reported | Not reported | Effective, pts value technology, convenient, savings in time and mileage driven, meets a digital preference, education at own pace | Effective | Cost to acquire equipment, staff training, may not be preferred modality | Cost | Timely, Effective, Efficient, Safe, and Patient-centered | Safe - Avoiding harm |  |
|  |  |  |  | Patients value technology |  | Staff training |  | Timely - Reduce wait times |  |
|  |  |  |  | Convenience |  | May not be preferred modality |  | Effective - Evidence based |  |
|  |  |  |  | Savings in time and mileage |  |  |  | Efficient - Lean |  |
|  |  |  |  | Meets a digital preference |  |  |  | Patient-centered - Respect autonomy |  |
|  |  |  |  | Education at own pace |  |  |  |  |  |
| Seib et al [54] | Not reported | Not reported | Effective, pts value technology, convenient, savings in time and mileage driven, meets a digital preference | Effective | Cost to acquire equipment, staff training, may not be preferred modality | Cost | Timely, Effective, Efficient, Safe, and Patient-centered | Safe - Avoiding harm |  |
|  |  |  |  | Patients value technology |  | Staff training |  | Timely - Reduce wait times |  |
|  |  |  |  | Convenience |  | May not be preferred modality |  | Effective - Evidence based |  |
|  |  |  |  | Savings in time and mileage |  |  |  | Efficient - Lean |  |
|  |  |  |  | Meets a digital preference |  |  |  | Patient-centered - Respect autonomy |  |
| Skvortsova et al [55] | Not reported | Not reported | Effective, pts value technology, convenient, savings in time and mileage driven, meets a digital preference | Effective | Cost to acquire equipment, staff training, may not be preferred modality | Cost | Timely, Effective, Efficient, Safe, and Patient-centered | Safe - Avoiding harm |  |
|  |  |  |  | Patients value technology |  | Staff training |  | Timely - Reduce wait times |  |
|  |  |  |  | Convenience |  | May not be preferred modality |  | Effective - Evidence based |  |
|  |  |  |  | Savings in time and mileage |  |  |  | Efficient - Lean |  |
|  |  |  |  | Meets a digital preference |  |  |  | Patient-centered - Respect autonomy |  |
| Stephenson et al [56] | Users reponded well to app | Satisfied | Effective, pts value technology, convenient, savings in time and mileage driven, meets a digital preference | Effective | Cost to acquire equipment, staff training, may not be preferred modality | Cost | Timely, Effective, Efficient, Safe, and Patient-centered | Safe - Avoiding harm |  |
|  |  |  |  | Patients value technology |  | Staff training |  | Timely - Reduce wait times |  |
|  |  |  |  | Convenience |  | May not be preferred modality |  | Effective - Evidence based |  |
|  |  |  |  | Savings in time and mileage |  |  |  | Efficient - Lean |  |
|  |  |  |  | Meets a digital preference |  |  |  | Patient-centered - Respect autonomy |  |
| Thesen et al [57] | Users reponded well to app | Satisfied | Effective, pts value technology, convenient, savings in time and mileage driven, meets a digital preference | Effective | Cost to acquire equipment, staff training, may not be preferred modality | Cost | Timely, Effective, Efficient, Safe, and Patient-centered | Safe - Avoiding harm |  |
|  |  |  |  | Patients value technology |  | Staff training |  | Timely - Reduce wait times |  |
|  |  |  |  | Convenience |  | May not be preferred modality |  | Effective - Evidence based |  |
|  |  |  |  | Savings in time and mileage |  |  |  | Efficient - Lean |  |
|  |  |  |  | Meets a digital preference |  |  |  | Patient-centered - Respect autonomy |  |
| Xia et al [58] | Users reponded well to app | Satisfied | Effective, pts value technology, convenient, savings in time and mileage driven, meets a digital preference | Effective | Cost to acquire equipment, staff training, may not be preferred modality | Cost | Timely, Effective, Efficient, Safe, and Patient-centered | Safe - Avoiding harm |  |
|  |  |  |  | Patients value technology |  | Staff training |  | Timely - Reduce wait times |  |
|  |  |  |  | Convenience |  | May not be preferred modality |  | Effective - Evidence based |  |
|  |  |  |  | Savings in time and mileage |  |  |  | Efficient - Lean |  |
|  |  |  |  | Meets a digital preference |  |  |  | Patient-centered - Respect autonomy |  |
|  |  |  |  |  |  |  |  |  |  |
|  |  |  |  |  |  |  |  |  |  |
| Zeng et al [59] | Users reponded well to app | Satisfied | Effective, pts value technology, convenient, savings in time and mileage driven, meets a digital preference | Effective | Cost to acquire equipment, staff training, may not be preferred modality | Cost | Timely, Effective, Efficient, Safe, and Patient-centered | Safe - Avoiding harm |  |
|  |  |  |  | Patients value technology |  | Staff training |  | Timely - Reduce wait times |  |
|  |  |  |  | Convenience |  | May not be preferred modality |  | Effective - Evidence based |  |
|  |  |  |  | Savings in time and mileage |  |  |  | Efficient - Lean |  |
|  |  |  |  | Meets a digital preference |  |  |  | Patient-centered - Respect autonomy |  |
| Zhang et al [60] | Not reported | Not reported | Effective, pts value technology, convenient, savings in time and mileage driven, meets a digital preference | Effective | Cost to acquire equipment, staff training, may not be preferred modality | Cost | Timely, Effective, Efficient, Safe, and Patient-centered | Safe - Avoiding harm |  |
|  |  |  |  | Patients value technology |  | Staff training |  | Timely - Reduce wait times |  |
|  |  |  |  | Convenience |  | May not be preferred modality |  | Effective - Evidence based |  |
|  |  |  |  | Savings in time and mileage |  |  |  | Efficient - Lean |  |
|  |  |  |  | Meets a digital preference |  |  |  | Patient-centered - Respect autonomy |  |
