# Supplementary material for: Analyzing the Effect of Telemedicine on Domains of Quality Through Facilitators and Barriers to Adoption: Systematic Review
Source: J Med Internet Res. 2023 Jan 5;25:e43601. doi: 10.2196/43601 (PMC9893735; doi:10.2196/43601)
Supplement: Multimedia Appendix 3 [file jmir_v25i1e43601_app3.docx]

**Appendix C:** Other observations incident to review

| Authors | Sample Size  (#s only) | Bias within study | Effect Size | Country of Origin (where was the study conducted?) | Statistics Used | Strength of Evidence | Quality of Evidence |  |
| --- | --- | --- | --- | --- | --- | --- | --- | --- |
| Bao et al [28] | 112 | One hospital in one country (selection bias), convenience sample and one ethnicity (sample bias) | not reprted | China | Descriptive statistics, Mann-Whitney U test, Wilcoxon test | I | A |  |
|  |  |  |  |  |  |  |  |  |
| Bendtsen et al [29] | 2129 | One country (selection bias), majority one sex (sample bias) | 2 mo (*OR*=0.85), 4 mo (*OR*=0.58), Cohen's *d*=0.25, small | Sweden | Descriptive statistics, negative binomial regression and maximum likelihood inference | I | A |  |
| Bhandari et al [30] | 200 | One country (selection bias), majority one race (sample bias) | not reprted | Nepal | Descriptive statistics, Chi-square test, *t*-tests | I | A |  |
| Catuara-Solarz et al [31] | 136 | One country only (selection bias), mostly male (sample bias) | Anxiety (*d*=0.32), Resilience (*d*=-0.48), Sleep (*d*=0.47),  Well-being (*d*=-0.41) | UK | Descriptive statistics, *t*-tests, Bonferroni correction, linear mixed models | I | A |  |
| Choi et al [32] | 180 | One country only (selection bias), mostly male (sample bias) | not reported | US | Descriptive statistics, McNemar tests, paired *t*-tests, Poisson regressions | I | A |  |
| Dalli et al [33] | 67 | One country only (selection bias), mostly male (sample bias) | not reported | Spain | Descriptive statistics, *t*-tests, Wilcoxon signed-rank test, Chi-squared test, Fisher's exact test, Mann-Whitney *U* test | I | A |  |
| do Amaral et al [34] | 400 | One country only (selection bias), mostly female (sample bias) | not reported | Brazil | Descriptive statistics, Chi-squared tests, Mann-Whitney, *t*-tests | I | A |  |
| Fernandez et al [35] | 1554 | One country only (selection bias), mostly female (sample bias) | completion of needed service (*OR*=1.38), Pap test (*OR*=1.56), and smoking cessation (*OR*=2.66), mammography (*OR*=1.53), colorectal cancer screening (*OR*=1.80), HPV vaccination of daughter (*OR*=1.61) | US | Descriptive statistics, Chi-squared tests, multivariate logistic regression | I | A |  |
| Guillaumier et al [36] | 399 | Six regions of one country (limited selection bias), mostly male (sample bias) | Reported no problems with personal care (*OR*=2.17) and usual activities (*OR*=1.66). | Australia | Descriptive statistics, median quartile regression, logistic regression, linear estimates and odds ratios, Fisher's exact test, and *t*-tests | I | A |  |
| Gustafson et al [37] | 310 | One country (selection bias), high % female and one ethnicity (sample bias) | medium (d=0.4) depression (*OR*=0.2, *p*=0.034), MH QOL (*OR*=0.32, *p*=0.007) | UK | Descriptive statistics, cumulative link mixed models | I | A |  |
| Huggins et al[38] | 111 | One country only (selection bias), mostly male (sample bias) | not reported | Australia | Descriptive statistics, linear mixed models | I | A |  |
| Itoh et al [39] | 99 | One country (selection bias), high % male and one ethnicity (sample bias) | not reported | Japan | Descriptive statistics, mixed-effects model for repeated measures | I | A |  |
| Jamali et al [40] | 43 | One country (selection bias), high % male and one ethnicity (sample bias) | not reported | Iran | Descriptive statistics, mixed-model repeated measures analysis of variance (ANOVA), Mann-Whitney signed-rank test | I | A |  |
| Leong et al [41] | 181 | One country (selection bias), high % male and one ethnicity (sample bias) | Health literacy (*OR*=2.80) | Taiwan | Descriptive statistics, Kolmogorov-Smirnov, Wilcoxon signed-rank test, paired *t*-tests | I | A |  |
| María Gómez et al [42] | 86 | One country (selection bias), high % male and one ethnicity (sample bias) | not reported | Columbia | Descriptive statistics, Shapiro Wilk’s test, *t*-tests | I | A |  |
| Mathiasen et al [43] | 86 | One country (selection bias), high % male and one ethnicity (sample bias) | not reported | Columbia | Descriptive statistics, Shapiro Wilk’s test, *t*-tests | I | A |  |
| Molavynejad et al [44] | 378 | One country only (selection bias), mostly one race (sample bias) | not reported | Iran | Descriptive statistics, *t*-tests | I | A |  |
| Morcillo-Muñoz et al [45] | 37 | One country only (selection bias), mostly one race and gender (sample bias) | not reported | Spain | Descriptive statistics, Mann-Whitney U test, Pearson Chi-square test, Fisher-Freeman-Halton, linear mixed models | I | A |  |
| Muschol et al [46] | 60 | One country only (selection bias), mostly one race and gender, convenience sample (sample bias) | not reported | Germany | Descriptive statistics, Mann-Whitney *U* test, Fisher exact test, Pearson's *r*, and Wilcoxon signed-rank test | I | A |  |
| Nagamitsu et al [47] | 217 | One country only (selection bias), mostly one race and gender (sample bias) | not reported | Japan | Descriptive statistics, Chi-square test | I | A |  |
| Ni et al [48] | 157 | One country only (selection bias), mostly one race and gender (sample bias) | not reported | China | Descriptive statistics, Chi-square and *t*-tests, Fisher exact test, Wilcoxon 2-sample tests | I | A |  |
| Pires et al [49] | 1936 | One country only (selection bias), mostly one race and gender (sample bias) | not reported | Bangladesh | Descriptive statistics, Chi-squared, and Fisher exact test | I | A |  |
| Pischke et al [50] | 204 | One country only (selection bias), mostly one race and gender (sample bias) | not reported | Germany | Descriptive statistics, linear mixed models | I | A |  |
| Roddy et al [51] | 506 | One country only (selection bias), mostly one race and gender (sample bias) | *b*=-0.47 | US | Descriptive statistics, ordinary least squares path analysis | I | A |  |
| Sahin et al [52] | 90 | One country only (selection bias), mostly one race (sample bias) | not reported | Turkey | Descriptive statistics, ANOVA, Tukey HDS test, Tamhanes T2 test | I | A |  |
| Sarker et al [53] | 126 | One country only (selection bias), mostly one race and gender (sample bias) | not reported | Bangladesh | Descriptive statistics, Mann-Whitney *U* test, ANOVA, *t*-tests | I | A |  |
| Seib et al[54] | 351 | One country only (selection bias), mostly one race and gender (sample bias) | Vitality (*d*=0.37), physical health (*d*=0.49) | Australia | Descriptive statistics, Chi-square tests, linear mixed models | I | A |  |
| Skvortsova et al [55] | 133 | One country only (selection bias), mostly one race and gender (sample bias) | *d*=0.61 | Netherlands | Descriptive statistics, *t*-test, Wilcoxon test, | I | A |  |
| Stephenson et al [56] | 424 | Single site in one country (selection bias), high % one gender and race (sample bias) | Safer sexual agreements (OR=1.87), discordant relationships (OR=0.62), breaking their sexual agreement (OR=0.51) | US | Descriptive statistics, Chi-square test, generalized estimating equations (GEE) | I | A |  |
| Thesen et al [57] | 161 | Single site in one country (selection bias), high % one gender and race (sample bias) | cardiac anxiety (*d*=0.38), health-related QoL (*d*=0.48) | Norway | Descriptive statistics, Kolmogorov-Smirnov, *t*-tests, Mann-Whitney *U* test. | I | A |  |
| Xia et al [58] | 156 | Single site in one country (selection bias), high % one gender and race (sample bias) | not reported | China | Descriptive statistics, Kolmogorov-Smirnov, *t*-tests, Mann-Whitney *U* test. | I | A |  |
| Zeng et al [59] | 300 | Single site in one country (selection bias), high % one gender and race (sample bias) | beta=4.198 | China | Descriptive statistics, Kolmogorov-Smirnov, Levene test, SEM, Chi-square tests, comparative fit index (CFI), Tucker-Lewis Index (TLI), root mean square approximation (RMSEA), weighted root mean square residual (WRMR) | I | A |  |
| Zhang et al[60] | 278 | Single site in one country (selection bias), high % one gender and race (sample bias) | not reported | China | descriptive statistics, Cho-square tests and *t*-tests | I | A |  |
